# The decline in the clinical relevance of pilocarpine and physostigmine monitored in pharmacology textbooks from 1878 to 2023: nine take-home messages for future (pharmacology) textbook authors

**DOI:** 10.1007/s00210-024-03558-x

**Published:** 2024-11-12

**Authors:** Laureen Ludwig, Roland Seifert

**Affiliations:** Institute of Pharmacology, Hannover Medical School, Carl-Neuberg-Straße 1, Hannover, 30625 Germany

**Keywords:** Pilocarpine, Physostigmine, Drugs, Textbook analysis, Content analysis, History of pharmacology

## Abstract

In a recent study, using hydrogen cyanide as paradigm, we have shown that pharmacological knowledge evolves non-linearly (https://pubmed.ncbi.nlm.nih.gov/38900251/). The aim of this study was to investigate the changes in the presentation of the drugs pilocarpine and physostigmine in textbooks from 1878 to 2023. The categories of structure, molecular mechanism of action, pharmacokinetics, effects, indications, adverse drug reactions, interactions, and contraindications were evaluated. The pharmacological knowledge on the molecular mechanism, chemical structure, and pharmacokinetics of pilocarpine and physostigmine changed the most during the period of 150 years. Until 1944, textbooks did not mention a molecular mechanism of action of pilocarpine and from 1951 onwards they described the activation of muscarinic acetylcholine receptors as the molecular basis of pilocarpine’s effect. Until 1944, most textbooks on physostigmine also did not mention the molecular mechanism of action. From 1951 onwards, the reversible inhibition of acetylcholinesterase is mentioned as the mechanism of action of physostigmine. In contrast, in the categories *effects*, *indications*, *adverse drug reactions*, *interactions*, and *contraindications*, the detected changes in the pharmacological knowledge presented were comparatively smaller. Older pharmacology textbooks were better than newer ones at discussing changes in knowledge and scientific errors. We noted substantial differences in the presentation of pilocarpine and physostigmine among German and US pharmacology textbooks. We show a decline of the clinical relevance of both drugs and their presentation in pharmacological textbooks with physostigmine being virtually irrelevant. But modern textbooks still discuss physostigmine substantially, fitting to studies on the obsolete drug reserpine (https://pubmed.ncbi.nlm.nih.gov/38103060/). Thus, textbooks often far lag clinical practice. Google Scholar conveys the incorrect impression that physostigmine is clinically more relevant than it is. An exponential decline in prescription numbers is a robust indicator of clinical obsolescence. From our study, we extract nine easily implementable take-home messages for future (pharmacology) textbook authors to ensure that this traditional teaching format will prevail against the competition of allegedly more “modern” teaching media.

**Supplementary Information:**

The online version contains supplementary material available at 10.1007/s00210-024-03558-x.

## Introduction

Pharmacological textbooks have a large influence on the knowledge of young physicians (Powell et al. 2014). Therefore, it is of concern that textbooks are substantially lagging clinical practice (Misera and Seifert 2023). In a recent study, using hydrogen cyanide as paradigm, we have shown that pharmacological-toxicological knowledge develops non-linearly (Ludwig and Seifert 2024). However, it is unknown whether the same type of non-linearity applies to all drugs. Therefore, in this case study, we assessed the presentation of pilocarpine and physostigmine based on sixteen German-language textbooks over three centuries, i.e., a similar period as for hydrogen cyanide (Ludwig and Seifert 2024). We additionally included a representative English-language textbook into our analysis. The long-term goal of these studies is to provide pharmacology professors with scientifically validated suggestions on how to write modern pharmacology textbooks that are actually read and appreciated by medical, pharmacy, and science students. Pharmacology textbooks must be reformed to avoid their extinction against the backdrop of other emerging teaching formats.

For interpretation of the history, current textbook knowledge on pilocarpine and physostigmine is presented below. Pilocarpine is an alkaloid derived from the leaves of the tropic plants *Pilocarpus jaborandi* and *Pilocarpus microphyllus* in South America (Komuro et al. 2023). By activating muscarinic acetylcholine receptors, it has a stimulating effect on cholinergic signaling and exerts a parasympathomimetic effect (Ritter et al. 2020). When used systemically, pilocarpine has effects on the heart and peripheral secretions, which are characterized by increased sweat, saliva, tear, and bronchial secretion and a negative chronotropic, dromotropic, and inotropic influence on the heart (Ritter et al. 2020; Aktories et al. 2022). Bradycardia and a drop in blood pressure are the result of its cardiac effects (Ritter et al. 2020). In addition, pilocarpine has an excitatory effect on the smooth muscles of the eye and thus causes miosis via contraction of the sphincter pupillae muscle and leads to near-accommodation and facilitated aqueous humor outflow via contraction of the ciliary muscle (Ritter et al. 2020). By facilitating aqueous humor outflow, which results from the dilation of the chamber angle and Schlemm’s canal, pilocarpine causes a reduction in intraocular pressure (Ritter et al. 2020; Aktories et al. 2022). Traditionally, pilocarpine has been used in ophthalmology as an miotic agent in the treatment of glaucoma, where pilocarpine eye drops are used even now (Jackson et al 2022; Brunton and Knollmann 2023). But with the introduction of newer drugs with greater efficacy and fewer adverse effects, like carboanhydrase inhibitors and beta-adrenoceptor antagonists, pilocarpine is no longer considered the first-line therapy for glaucoma (Berufsverband der Augenärzte Deutschlands e.V., 2006; Ostler et al. 2021; Wagner et al. 2022). In 2021, pilocarpine hydrochloride ophthalmic solution was approved for the use in adults with presbyopia (Jackson et al. 2022). Furthermore, oral 5 mg pilocarpine four times daily (20 mg/d) is used to relieve xerostomia that followed radiotherapy of the head and neck or in patients with Sjögren’s syndrome (Vivino 1999; Komuro et al. 2023). Pilocarpine is also used in the diagnosis of cystic fibrosis (Seifert 2019). Possible adverse effects of pilocarpine include colicky abdominal pain, a drop in blood pressure, and bronchoconstriction, which in turn can lead to asthmatic attacks (Ritter et al. 2020; Aktories et al. 2022).

Physostigmine is an alkaloid isolated from the Calabar beans or ordeal beans and is a short-acting reversible cholinesterase inhibitor (Boroughf 2016; Seifert 2019; Brunton and Knollmann 2023). Through its reversible inhibition of acetylcholinesterase and the less specific butyrylcholinesterase, physostigmine increases the concentration of acetylcholine in the synaptic cleft and thus enhances cholinergic signaling (Seifert 2019; Ritter et al. 2020). Physostigmine, as a tertiary ammonium compound, shows a permeability through the blood-brain barrier unlike other cholinesterase inhibitors such as neostigmine, a quaternary compound (Arens and Kearney 2019; Ritter et al. 2020). Previously, physostigmine was used for the treatment of myasthenia gravis, but later superseded by neostigmine (Katz and Bahohn 2021). In the past, physostigmine had been tested as a therapy in the early stages of Alzheimer’s disease to improve cognitive impairment caused by a cholinergic deficit (Drachman and Leavitt 1974; Bartus et al. 1982; Goodman 1985). However, later studies have not found any significant effect of physostigmine in the symptomatic treatment of Alzheimer’s patients (Coelho Filho and Birks 2001). Due to its permeability through the blood-brain barrier, physostigmine was used in central anticholinergic syndrome, which can be caused by atropine, some antipsychotic drugs, or first-generation histamine H_1_-receptor antagonists, for example (Seifert 2019; Ritter et al. 2020; Hölle et al. 2023). Furthermore, local administration of physostigmine in the form of eye drops was used in the treatment of glaucoma, as physostigmine leads to miosis and improved aqueous humor outflow through contraction of the ciliary muscle (Snyder 1964; Realini, 2011; Ritter et al. 2020; Brunton and Knollmann 2023). However, in modern guidelines for glaucoma treatment, physostigmine does not play a role anymore (Marshall et al. 2018). Nowadays, the clinical use of physostigmine is very restricted, but physostigmine preparations are still manufactured and used in the USA (Bongoni 2020). Adverse drug reactions that can occur because of physostigmine administration include bradycardia, vomiting, diarrhea, urinary urgency, excessive secretion of sweat, saliva, bronchial, and gastric juices, as well as constriction of the bronchi, which can lead to asthmatic attacks (Seifert 2019; Ritter et al. 2020; Aktories et al. 2022). In addition, an overdose of physostigmine can induce a neuromuscular blockade (depolarization block), which leads to paralysis (Ritter et al. 2020). In the event of physostigmine intoxication, atropine is conceptually suitable as a partial antidote (Brunton and Knollmann 2023).

The aim of this study was to investigate the changes in the presentation of the drugs pilocarpine and physostigmine in sixteen German-language pharmacology textbooks from 1878 to 2020 and several modern editions of the US pharmacology textbook *Goodman & Gilman’s: The Pharmacological Basis of Therapeutics.* The German prescription figures and international PubMed entries on the drugs were also considered to analyze the change in the clinical relevance of pilocarpine and physostigmine and compare it with the presentation in the textbooks. This should contribute to determine whether the presentation of pilocarpine and physostigmine in the textbooks corresponds to the reality of prescribing and, if this is not the case, how pharmacology textbooks should be adapted to convey practice-relevant and up-to-date correct content to medical students and practicing physicians.

## Material and methods

### Selection of textbooks

One textbook per decade was analyzed as an example to compare the content presented for pilocarpine and physostigmine in pharmacological textbooks from 1878 onwards (see Table 1). The selection criteria included that the textbooks must be aimed at medical students and doctors and published in German language. A further selection criterion was the availability of the textbooks.

**Table 1:** Pharmacology textbooks used for the analysis. All textbooks analyzed are listed with the names of the authors, edition, reference, total number of pages, and page references from the index relating to pilocarpine and physostigmine. The relative share of the active substance pages in the total number of pages of the textbook is also indicated. If a page is listed in the index, but pilocarpine or physostigmine are not explicitly dealt with on the corresponding page, the page number is not listed in the table. The term “explicitly dealt with” refers to the naming of pilocarpine and physostigmine on the corresponding page. Pages on which content about the active substances is continued from preceding or neighboring pages are also listed as individual pages in the table. This also applies to pages that are labelled as subsequent pages in the index. Only the pages directly surrounding the page specified in the index were checked for a continuation of the content. A continuation of the content on pages not listed in the index was only considered to be one if the drug is mentioned by name and specific information regarding pilocarpine or physostigmine is discussed on the page. Comparisons and references to the drugs do not count

ID-Number	Textbook group	Author	Year of publication	Edition	Reference	Pages	Drug-related pages (keyword index)	
							Pilocarpine	Physostigmine
1	1	Buchheim	1878	3	Buchheim (1878)	618	490, 491, 492	492, 493, 494, 495
2	1	Schmiedeberg	1883	1	Schmiedeberg (1883)	279	64, 65, 66, 67, 68	69, 70, 71, 72, 73
3	1	Husemann	1892	3	Husemann (1892)	705	636, 652, 653, 654, 655, 656	484, 485, 486, 487
4	1	Filehne	1901	10	Filehne (1901)	421	29, 30, 31, 66, 71, 77, 258, 288, 295, 299, 300, 301, 385	8, 29, 30, 66, 77, 78, 79, 258, 299, 300
5	2	Tappeiner	1919	13	Tappeiner (1919)	499	208, 209, 303, 304	305, 306
6	2	Schmiedeberg	1921	8	Schmiedeberg (1921)	657	188, 189, 190, 193, 194, 195	186, 204, 205, 206, 207, 208, 209
7	2	Hoesslin, Mueller	1933	4	Hoesslin and Mueller (1933)	245	26, 29, 33, 96, 97, 98, 99, 103, 107	29, 82, 88, 96, 97, 102, 103
8	2	Eichholtz	1944	3 and 4	Eichholtz (1944)	525	100, 230, 234, 235, 248, 324, 360	230, 233, 234, 235, 236, 248, 360
9	3	Eichholtz	1951	7	Eichholtz (1951)	594	35, 107, 197, 253, 254, 255, 315, 350, 363	18, 124, 200, 250, 252, 253, 254, 255, 271, 273
10	3	Kuschinsky, Luellmann	1964	1	Kuschinsky and Luellmann (1964)	331	6, 7, 8	8, 9
11	3	Forth, Henschler, Rummel	1977	2	Forth et al. (1977)	672	80, 81, 82, 83, 84	80, 81, 82, 83, 84, 422
12	3	Estler	1986	Supp. study edition	Estler (1986)	648	33, 34, 35, 38, 586	35, 36, 38, 526
13	4	Oberdisse, Hackenthal, Kuschinsky	1997	1	Oberdisse et al. (1997)	770	81	81, 82
14	4	Aktories, Förstermann, Hofmann, Starke	2005	9	Aktories et al. (2005)	1189	45, 148, 149, 168, 169	155, 165, 166, 170, 971, 1076
15	4	Graefe, Lutz, Bönisch	2016	2	Graefe et al. (2016)	836	109, 110, 111	112, 113, 316, 729, 730, 737, 745, 755
16	4	Freissmuth, Offermanns, Böhm	2020	3	Freissmuth et al. (2020)	1064	264, 265, 450	265, 266

### Analyzing the data

**Fig. 1:**
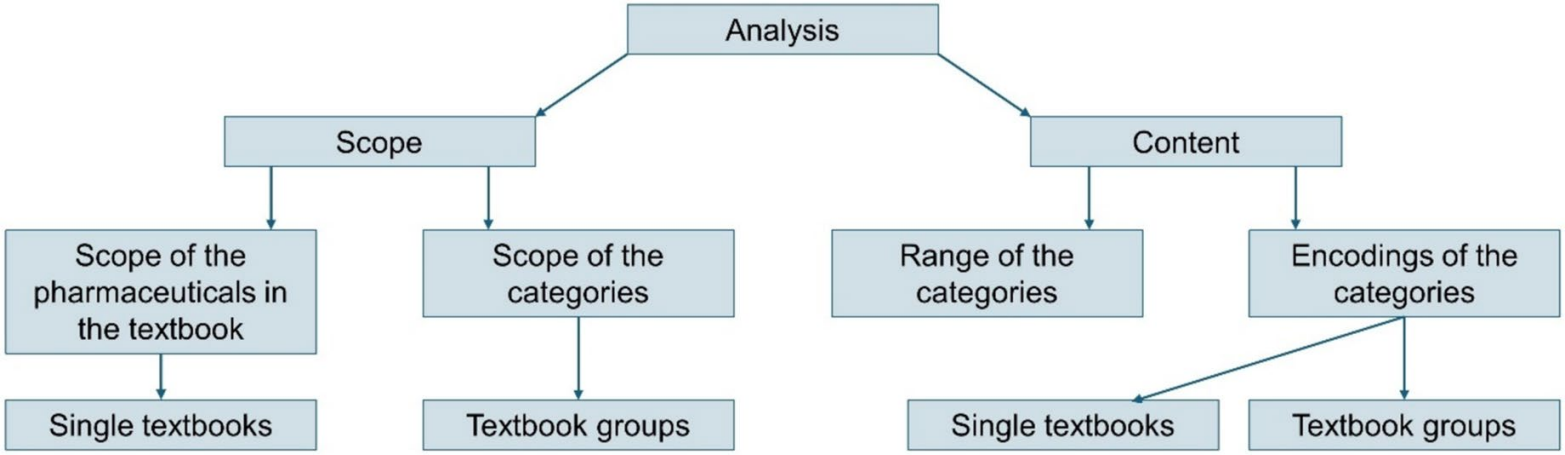
Methodological procedure for analyzing the data. For a detailed list of the encodings, see supplemental Tables S1–S16

Figure 1 illustrates the methodological approach used to analyze the data. Tables S1–S16 show the analysis categories with encodings and the detailed results for the textbook groups. The scope of pilocarpine- and physostigmine-related pages in the textbooks was analyzed. The scope of the categories (structure, molecular mechanism of action, pharmacokinetics, effects, indications, adverse drug reactions, interactions, and contraindications) was determined. The textbooks were divided into textbook groups chronologically: *1878–1901 (textbook group 1)*, *1919–1944 (textbook group 2)*, *1951–1986 (textbook group 3)*, and *1997–2020 (textbook group 4).*

The listed content of the textbooks was also analyzed for both drugs. All content relating directly or indirectly to the drug was evaluated. Content relating to the use and effects of the drug in animals was not analyzed unless the author described its validity for humans. For the categories *molecular mechanism of action*, *pharmacokinetics*, *effects*, *indications*, *adverse drug reactions*, *interactions*, and *contraindications*, the similarity of content between pilocarpine and physostigmine was also compared (see supplemental Figs. S1–S7). The average range of the categories—based on the range of all encodings within the category—was calculated.

An inductive approach was chosen to digitize the data. This procedure makes it possible, after the verbatim transfer of the primary information from the textbooks into the associated categories and subsequent definition of the encodings, to present all the collected content in a comparable form. All encodings within an analysis category were assigned numbers, which in turn were assigned to the textbooks that listed the contents of the corresponding encodings.

### Drug prescriptions

The number of drug prescriptions for pilocarpine and physostigmine from 2003 to 2023 was analyzed. The data is based on the prescription figures of the Scientific Institute of the AOK (WIdO) and includes all prescriptions for patients with statutory health insurance (GKV) in Germany. For pilocarpine, prescriptions of preparations with ATC codes N07AX01 and S01EB01 were analyzed in total. From 2016 onwards, formulations with pilocarpine are also included in the prescription figures. For physostigmine, prescriptions of preparations with ATC code V03AB19 were analyzed.

### PubMed and Google Scholar search results

The number of PubMed and Google Scholar search results for pilocarpine and physostigmine from 1878 to 2020 was analyzed. No filters were applied to the PubMed and Google Scholar search in terms of text availability, article attribute, article type, or language. Patents and citations were not included in the Google Scholar search.

Due to the increased inclusion of predatory content in the Google Scholar search results and thus reduced scientific reliability, the data about Google Scholar is evaluated exclusively as a supplement to the PubMed search results.

### Comparison with US pharmacology textbooks

An exemplary analysis of four editions of *Goodman & Gilman’s: The Pharmacological Basis of Therapeutics* from 1996, 2006, 2018, and 2023 was made. The textbooks Hardman et al. (1996), Brunton et al. (2006), Brunton et al. (2018) and Brunton and Knollmann (2023) were analyzed. The editions were selected based on the publication years of the German textbooks in the fourth textbook group. The results were compared to the German textbooks in the fourth textbook group. The US textbooks were evaluated regarding the scope of the drugs in the textbooks, the indications given, and the discussion of outdated content.

## Results and discussion

### Scope of drug-related content in the German-language textbooks

**Fig. 2:**
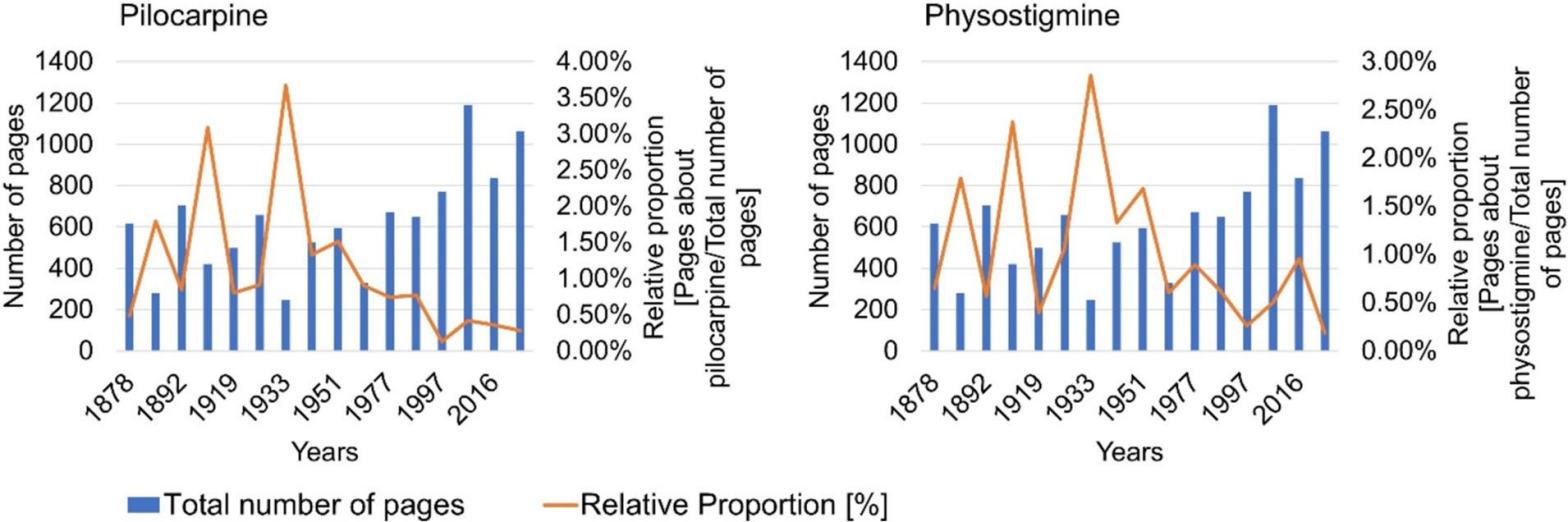
Total number of pages in the textbooks and relative proportion of active substance pages in the total number of pages in the textbook. The left panel shows the data for pilocarpine and the right panel for physostigmine

Figure 2 shows the total number of pages of the textbooks and the relative share of the active substance pages in the total number of pages of the textbook. Supplemental Fig. S8 shows the number of pages with content on pilocarpine and physostigmine.

The number of pages with content on pilocarpine increased constantly in the period 1878–1901 until it reached its maximum in textbook 4 in 1901 with a total of 13 pages (see supplemental Fig. S8). The number of active substance pages then fell until it reached its minimum in textbook 13 in 1997 with one active substance page. In the period 1997–2020, the number of pages with content on the active ingredient remained at a low-medium level. The relative proportion of active substance pages in the total number of pages in the textbooks in the period 1878–1933 ranged from 0.49% in textbook 1 in 1878 to 3.09% in textbook 4 in 1901 and reached its maximum in textbook 7 in 1933 with a value of 3.67% (see Fig. 2). The relative proportion then dropped significantly to 1.33% in textbook 8 in 1944. In the period 1944–1997, a further reduction in the percentage of active ingredient pages in the total number of textbook pages could be recognized until the minimum of 0.13% was reached in textbook 13 in 1997.

The absolute number of textbook pages dealing with physostigmine peaked in textbook 4 in 1901 and textbook 9 in 1951 with 10 pages each (see Fig. 2). Then, the number of pages dropped significantly and reached its minimum in textbook 10 in 1964, in textbook 13 in 1997, and in textbook 16 in 2020, each with two active substance pages. The relative proportion of textbook pages mentioning physostigmine out of the total number of textbook pages was maximized in textbook 7 in 1933 with 2.86%. Further peaks could be found in textbook 2 in 1883 with a percentage share of 1.79%, in textbook 4 in 1901 with a share of 2.38%, and textbook 9 in 1951 with a value of 1.68%. In the period 1964–2020, the relative proportion decreased significantly and reached the minimum in textbook 16 in 2020 with a value of 0.19%.

### Range of categories

**Fig. 3:**
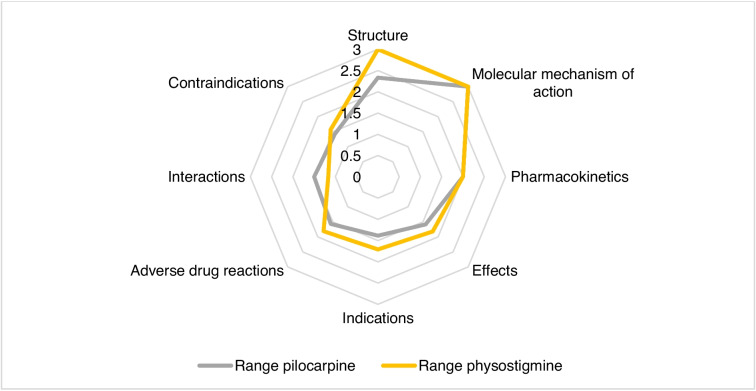
Range of all categories for pilocarpine and physostigmine. The calculation of a category’s range is based on the average range of all encodings within this category. The range of individual encodings results from the difference between the highest number of entries per textbook group and the lowest number. The number of possible entries and therefore the range of an encoding varies between 0 and 4. This is based on the number of textbooks per textbook group

Figure 3 shows the range of the categories. The average range of all categories of pilocarpine was 1.85. The categories *molecular mechanism of action*, *structure*, and *pharmacokinetics* showed an above-average range (see Fig. 3). A below-average range was found in the categories *effects*, *adverse drug reactions*, *interactions*, *contraindications*, and *indications*. For physostigmine, all analyzed categories had a range of 2.01 on average. An above-average range was found in the categories *molecular mechanism of action* and *structure.* The categories *effects*, *adverse drug reactions*, *indications*, *interactions*, and *contraindications* showed below-average ranges. The range of the category *pharmacokinetics* can be considered average. For both pilocarpine and physostigmine, the categories *molecular mechanism of action*, *structure*, and *pharmacokinetics* showed the three largest ranges. Thus, the change in knowledge in the period 1878–2020 regarding the molecular mechanism of action, structure, and pharmacokinetics of pilocarpine and physostigmine was most pronounced compared to all categories analyzed. Comparatively smaller changes in knowledge were found in the categories *effects*, *adverse drug reactions*, *indications*, *interactions*, and *contraindications* for both drugs*.*

### Structure

**Fig. 4:**
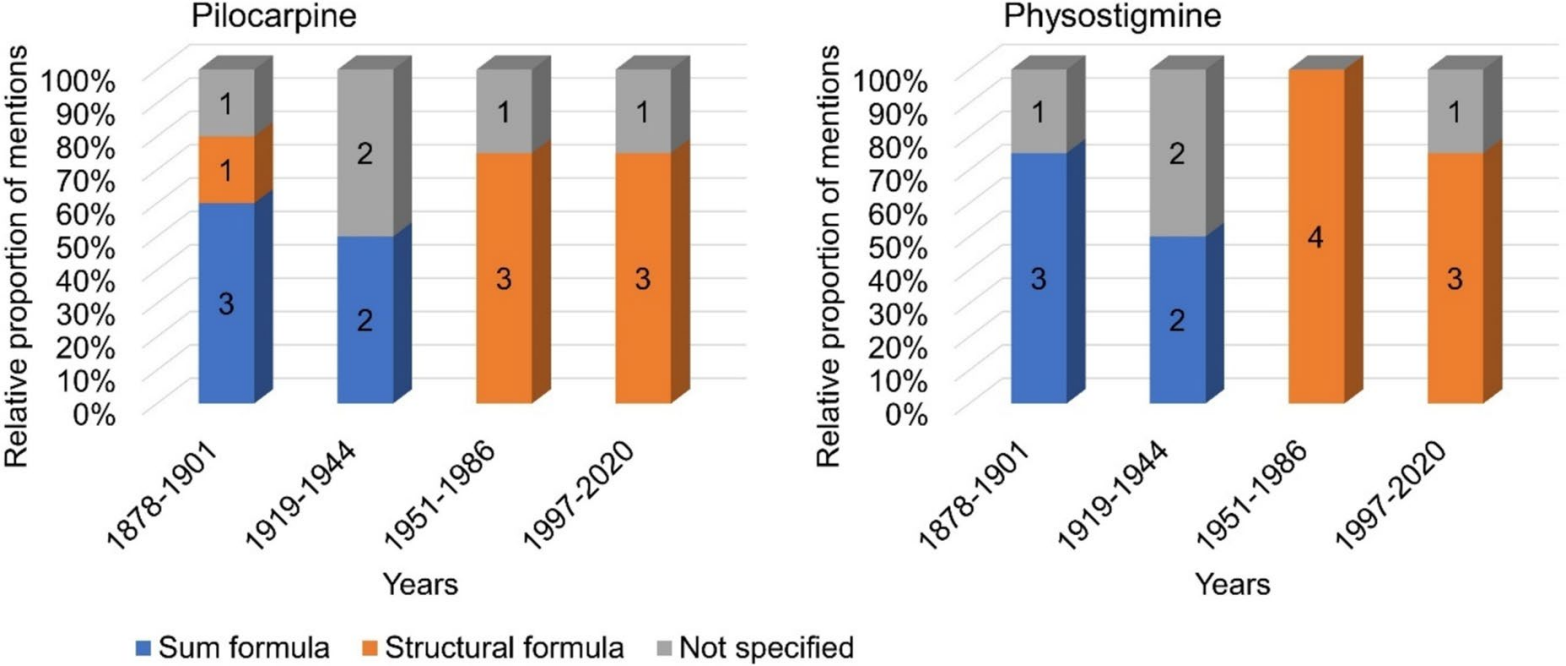
Information on the structure of active substances. The left panel shows the entries for pilocarpine and the right panel for physostigmine. The number of entries per encoding is given as a percentage of the total number of entries per textbook group

Figure 4 shows the information on the structure of pilocarpine and physostigmine according to textbook groups. In the first textbook group, 75% of the textbooks described the sum formula of pilocarpine (see Fig. 4). One textbook in the first textbook group listed both the sum formula and structural formula (textbook 4). In the second group of textbooks, the sum formula was found in 50% of the textbooks. Seventy-five percent of the textbooks in the third and fourth textbook groups listed the structural formula of the active substance. For physostigmine, 75% of the textbooks in the first textbook group and 50% of the books in the second textbook group listed the sum formula of the drug. In the third and fourth textbook groups, 100% and 75% of the textbooks respectively showed the structural formula of physostigmine.

Thus, the presentation of both drugs’ molecular structure changes dramatically from the second to the third textbook group. Knowledge of the structural formula of physostigmine changed considerably from 1951 onwards, whereas the structural formula of pilocarpine was already known from the first textbook group.

### Molecular mechanism of action

Supplemental Fig. S9 shows the number of molecular mechanisms of action listed for pilocarpine and physostigmine per textbook group. For pilocarpine, 100% of the textbooks in the first and second textbook group did not list any content on the molecular mechanism of action (see supplemental Fig. S9). In the third and fourth textbook groups, 50% and 100% of the textbooks respectively mentioned activation of muscarinic acetylcholine receptors as the molecular mechanism of action of pilocarpine. In the case of physostigmine, 100% of the textbooks in the first textbook group did not describe a molecular mechanism of action of the active substance. In the second group of textbooks, 75% of the textbooks did not describe the mechanism of action of the drug and 25% mentioned the inhibition of acetylcholinesterase without specifying the inhibition process. In the third and fourth textbook groups, the reversible inhibition of acetylcholinesterase was listed most frequently and was described as the mechanism of action of physostigmine by 75% of the textbooks in the third textbook group and 100% of the textbooks in the fourth textbook group.

Accordingly, the molecular mechanism of action of physostigmine was discovered and portrayed in the textbooks earlier than pilocarpine’s mechanism of action. Overall, there is a significant increase in knowledge regarding the molecular mechanisms of action of the drugs for both pilocarpine and physostigmine from 1951 onwards.

### Pharmacokinetics

**Fig. 5:**
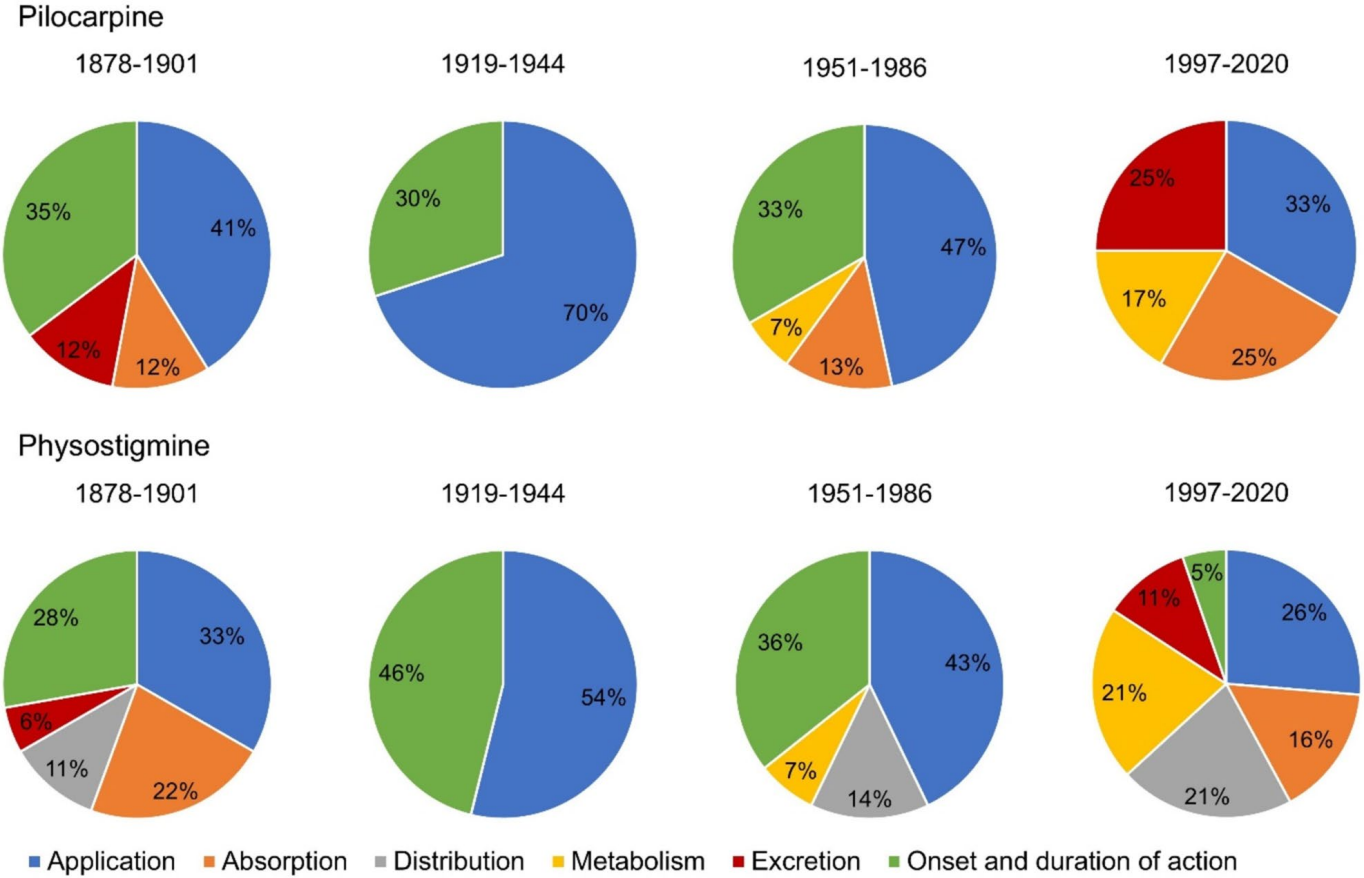
Information on the pharmacokinetic details of the drug. The upper panel shows the content for pilocarpine and the lower panel for physostigmine. The relative number of entries in the total number of analyzed entries is shown. Textbooks that did not contain any information on the pharmacokinetics of the drugs are not shown and can be found in the supplemental tables S5 and S6. The breakdown of the listed contents on the pharmacokinetics of the active substances is based on the ADME model of absorption, distribution, metabolism, and excretion. For the sake of completeness, information regarding the therapeutic applications of the drugs and the onset and duration of action were also evaluated. Under *application*, only content on the drug’s applications that are applied as part of the therapeutic use of the drug is listed. In *onset and duration of action*, only content relating to specific dates of the onset and duration of the drug action is considered. In *distribution*, only specific mentions of penetration of the blood-brain barrier and CNS permeability are included int the corresponding encoding

Figure 5 shows the contents about the pharmacokinetics of pilocarpine and physostigmine according to textbook groups. In the case of pilocarpine, the focus in all four textbook groups was on the application of the drug. However, the number of contents per textbook group relating to the application of pilocarpine decreased from the second to the fourth textbook group. Information on the onset and duration of action of pilocarpine was provided exclusively by textbooks in the first to third textbook group. In the fourth textbook group, and thus from 1997 onwards, no more content could be assigned to the subcategory *onset and duration of action*. In contrast, the quantity of information on excretion, absorption, and metabolism of the active substance was significantly higher in the fourth textbook group compared to the first to third textbook group.

In the case of physostigmine, as with pilocarpine, the focus in all four textbook groups was on the application of the drug. A decrease in the content relating to the subcategory *application* per textbook group could be observed from the second to the fourth textbook group, too. Content on the onset and duration of action of physostigmine was provided by textbooks in all four textbook groups, but the number of contents in the subcategory *onset and duration of action* decreased drastically from the second to the fourth textbook group. The quantity of content on distribution, excretion, and metabolism of the active substance in the fourth textbook group was significantly higher than in the first to third textbook groups.

Overall, there is a decreasing focus on the application and the onset and duration of action for both drugs from 1997 onwards and instead an increasing presentation of content relating to the absorption, distribution, excretion, and metabolism of the drugs. This illustrates the decreasing practical relevance of pilocarpine and physostigmine and the associated increase in the focus of modern pharmacology textbooks on more complex and less practical aspects of pharmacokinetics.

### Effects

**Fig. 6:**
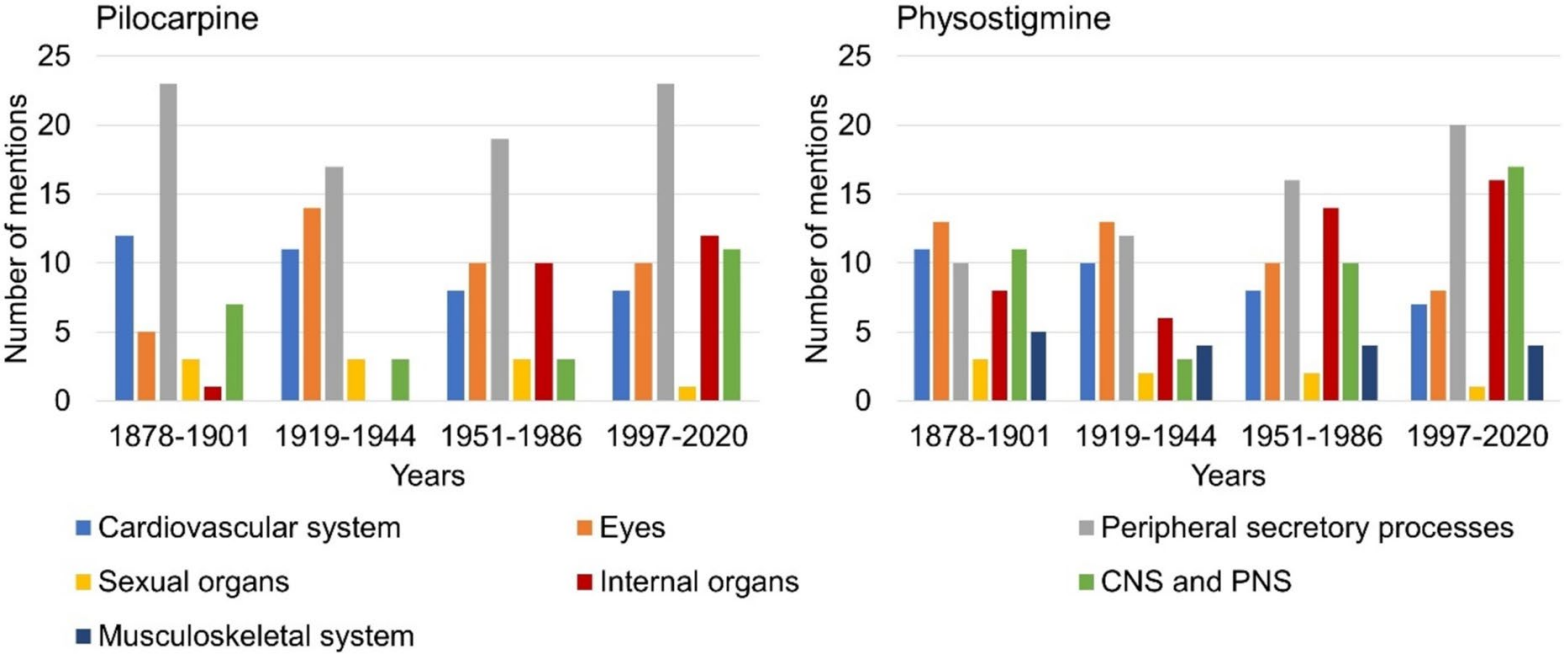
Information on the effects of the drugs. The left panel shows the entries for pilocarpine and the right panel for physostigmine. The absolute number of entries is shown

Figure 6 shows the information on the effects of pilocarpine and physostigmine per textbook group. In the case of pilocarpine, an increase in the scope of the effects listed could be seen from the second to fourth textbook group (see supplemental Table S7). In all four textbook groups, the focus was on drug effects that affect peripheral secretion processes. For physostigmine, an increase in the scope of described drug effects could be identified from the second to fourth textbook group (see supplemental Table S8). The textbooks of the first and second textbook group described effects of the drug that are related to the eye the most. The textbooks in the third and fourth textbook groups focused on physostigmine effects that affect peripheral secretions. Effects on the musculoskeletal system were only mentioned in relation to physostigmine.

**Fig. 7:**
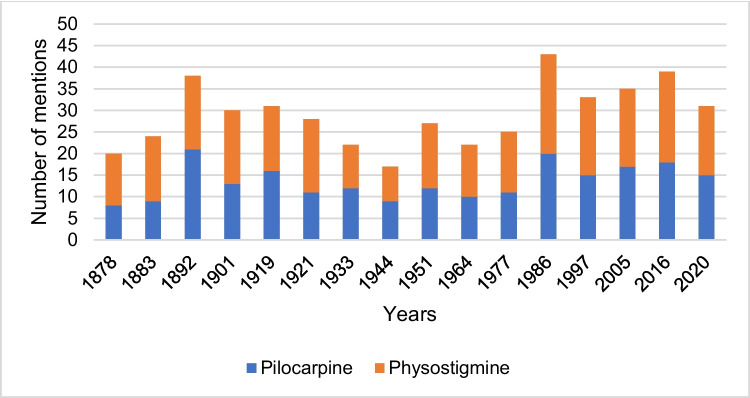
Number of effects mentioned for pilocarpine and physostigmine according to individual textbooks

Figure 7 demonstrates how many effects per textbook are described for pilocarpine and physostigmine in the period 1878–2020. Overall, no large changes regarding the described effects were observed during the entire study period for both drugs (see Fig. 7).

### Indications

**Fig. 8:**
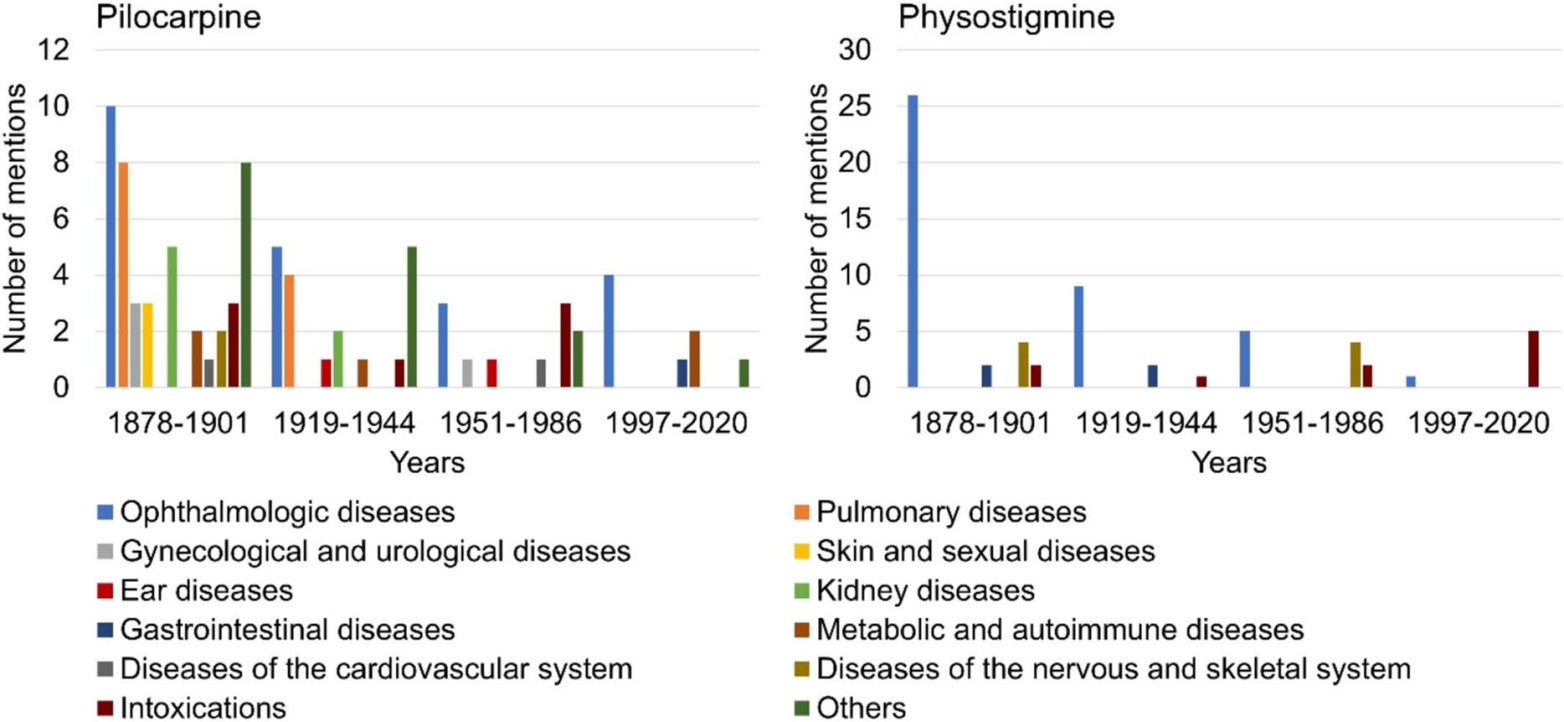
Information on the indications of the active substances. The contents for the indications of pilocarpine are shown in the left panel, the entries for the indications of physostigmine are shown in the right panel. The absolute amount of information is shown

Figure 8 shows the listed indications of pilocarpine and physostigmine according to textbook groups. The textbooks in the first textbook group listed ten different indications for pilocarpine (see Fig. 8). The textbooks in the second and third textbook groups each listed six indications for the drug. In the fourth group of textbooks, four different indications for pilocarpine were described. Therefore, the number of listed pilocarpine indications drastically decreased from 1878 to 2020. In all four textbook groups, ophthalmological diseases were among the most frequently listed indications for the drug. From 1997 to 2020, only ophthalmological, gastrointestinal and metabolic and autoimmune diseases as well as others like the increase of saliva secretion were mentioned as indications for pilocarpine.

For physostigmine, the textbooks in the first textbook group listed four different indications. The second and third textbook groups each described three indications for the active substance. The textbooks in the fourth textbook group listed two different indications for physostigmine. In comparison with pilocarpine, the number of indications mentioned for physostigmine was always lower. This indicates that the medical relevance and use of pilocarpine were always higher than that of physostigmine. In the first, second, and third textbook groups, ophthalmological diseases were the most common indications for physostigmine. In the fourth textbook group, the focus was on the treatment of intoxications with physostigmine. From 1997 onwards, only ophthalmological diseases and intoxications were still listed as indications for physostigmine.

**Fig. 9:**
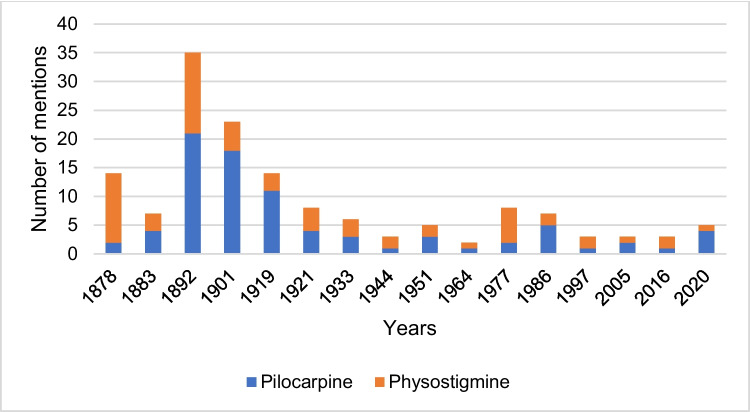
Number of indications mentioned for pilocarpine and physostigmine according to individual textbooks

Figure 9 shows how many indications per textbook are described for pilocarpine and physostigmine in the period 1878–2020. From 1892 to 2020, there was an exponential drop in the number of pilocarpine and physostigmine indications listed (see Fig. 9). The only exception to this trend for physostigmine was textbook 11 in 1977 with six listed indications. Overall, this shows a decrease in the listed indications and areas of application for both drugs by 2020.

Supplemental Fig. S10 shows the total number of indications mentioned for pilocarpine and physostigmine per textbook group. The change in the listed indications of the drugs could be approximated by the exponential regression of the equation *y* = 355.57e−0.553x with a coefficient of determination of R=0.9491 (see supplemental Fig. S10). The regression underlines the previously described exponential decline in pilocarpine and physostigmine indications in the 1878–2020 study period. This shows that the therapeutic importance and use of the drugs have decreased over time and that the two drugs are now less relevant in the medical context.

### Adverse drug reactions

**Fig. 10:**
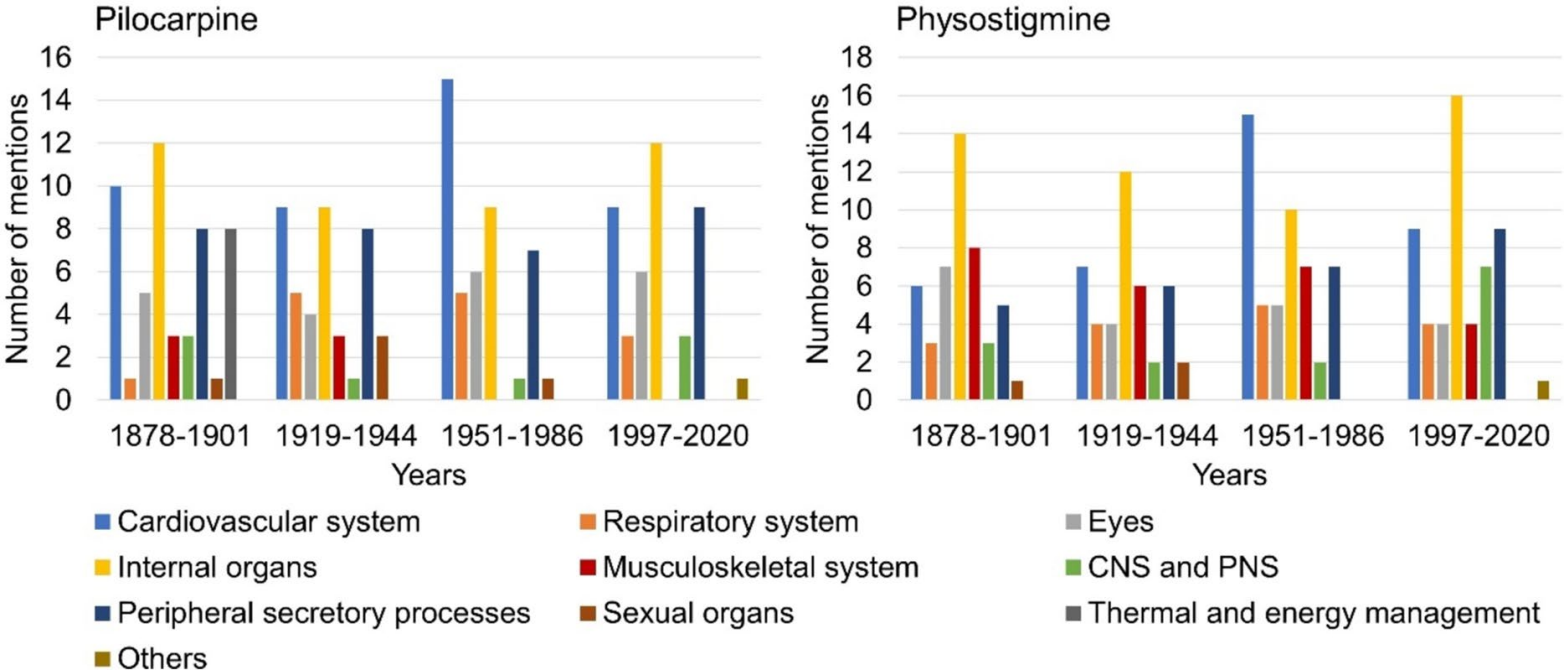
Information on adverse drug reactions of the r active substances. The left panel shows the content on the ADRs of pilocarpine. The right panel shows the entries for physostigmine. The absolute number of entries is shown

Figure 10 shows the information on the adverse drug reactions of pilocarpine and physostigmine. The amount of content on the adverse drug reactions of pilocarpine decreased from the first to the second textbook group (see supplemental Table S11). The textbooks in the first textbook group described side effects from nine different systems. The information in the second textbook group could be divided into eight systems. The textbooks of the third and fourth textbook group listed adverse drug reactions from seven different systems each. Adverse drug reactions affecting the thermal and energy balance were only mentioned in relation to pilocarpine. In the case of physostigmine, an increase in the amount of content on adverse drug reactions could be observed from 1919 onwards (see supplemental Table S12). The textbooks in the first, second, and fourth textbook groups listed side effects that could each be assigned to eight systems. In the third textbook group, the information referred to seven different systems.

Supplemental Fig. S11 shows how many adverse drug reactions per textbook are described for pilocarpine and physostigmine in the period 1878–2020. Overall, no significant quantitative changes regarding the described ADRs were observed during the entire study period for both drugs (see supplemental Fig. S11). This shows that knowledge about the adverse drug reactions of pilocarpine and physostigmine was already available at an early stage. This suggests that at the beginning of the period, the drugs were used despite the known adverse effects, primarily the drugs were used due to the lack of therapeutic alternatives and the number of indications mentioned was correspondingly high. However, as the number of alternative treatment options increased, the clinical relevance of the of the drugs declined.

### Interactions

**Fig. 11:**
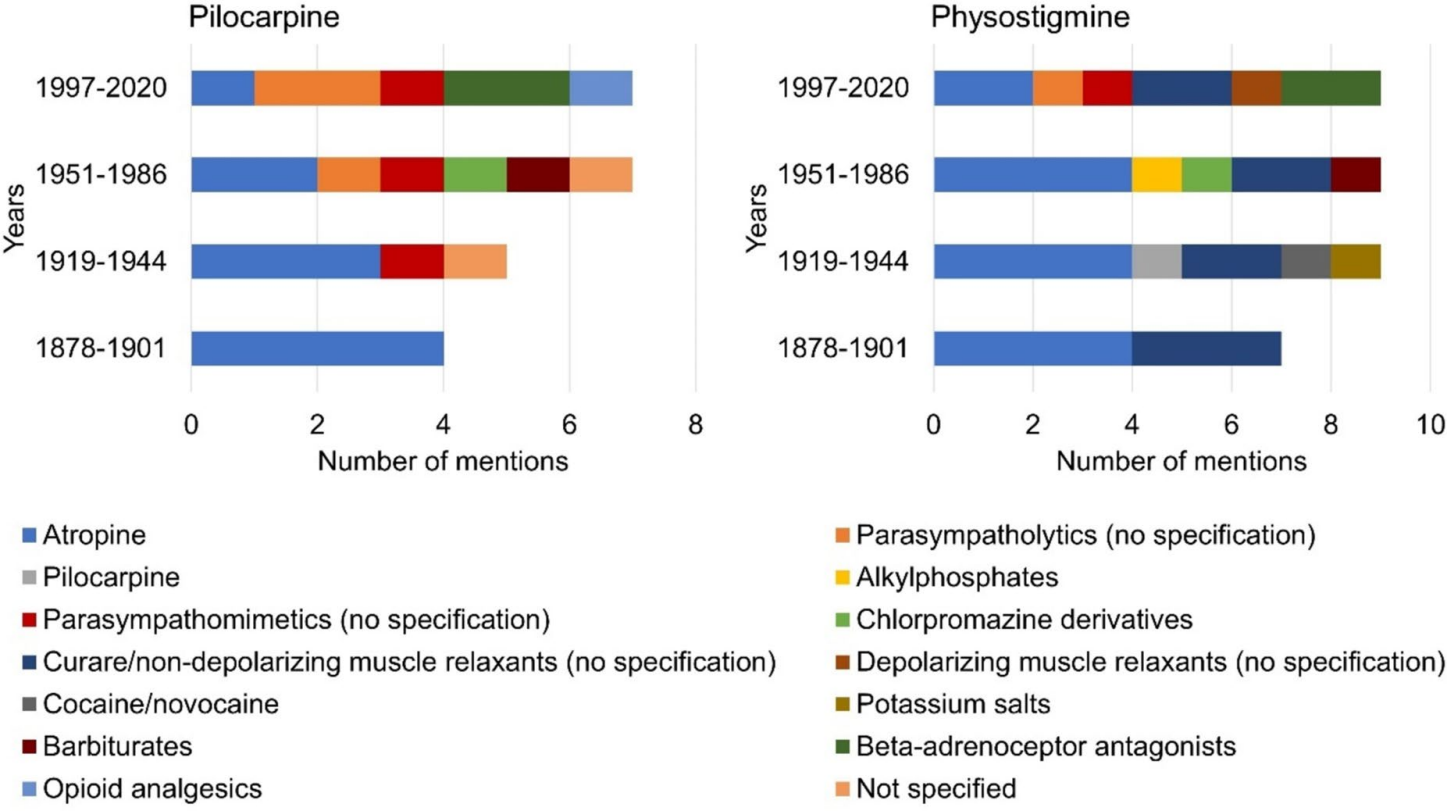
Information on interactions of pilocarpine and physostigmine with other drugs*.* The left panel shows the information on pilocarpine and the right panel the information on physostigmine. The absolute amount of information is shown

Figure 11 shows the named interactions of the drugs according to textbook groups. The textbooks of the first textbook group mentioned one interaction of pilocarpine with other medicinal products (see Fig. 11). The textbooks in the second textbook group provided information on two different interactions. The information in the third and fourth group of textbooks referred to five different interactions each. In the first, second, and third textbook groups, interactions of pilocarpine with atropine were described most frequently. The textbooks in the fourth group most frequently described interactions of pilocarpine with parasympatholytics and beta-adrenoceptor antagonists.

The textbooks of the first textbook group named two different drug interactions of physostigmine. The entries in the second and third textbook groups could each be assigned to five different interactions. The fourth textbook group listed six interactions with other drugs for physostigmine. The interaction with atropine was the most frequently mentioned drug interaction of physostigmine in the first, second, and third textbook groups. The textbooks in the fourth textbook group most frequently described interactions of the active substance with atropine, curare, and other non-depolarizing muscle relaxants as well as beta-adrenoceptor antagonists. Therefore, interactions of both pilocarpine and physostigmine with atropine were known since the beginning of the study period in 1878. However, due to the increase in the number of interactions mentioned from 1878 to 2020, there was a reduced focus on the interaction with atropine until 2020. Overall, this shows an increase in pharmacological knowledge regarding the interactions of pilocarpine and physostigmine by 2020 and therefore a heterogenization of the interactions described for both drugs.

### Contraindications

**Fig. 12:**
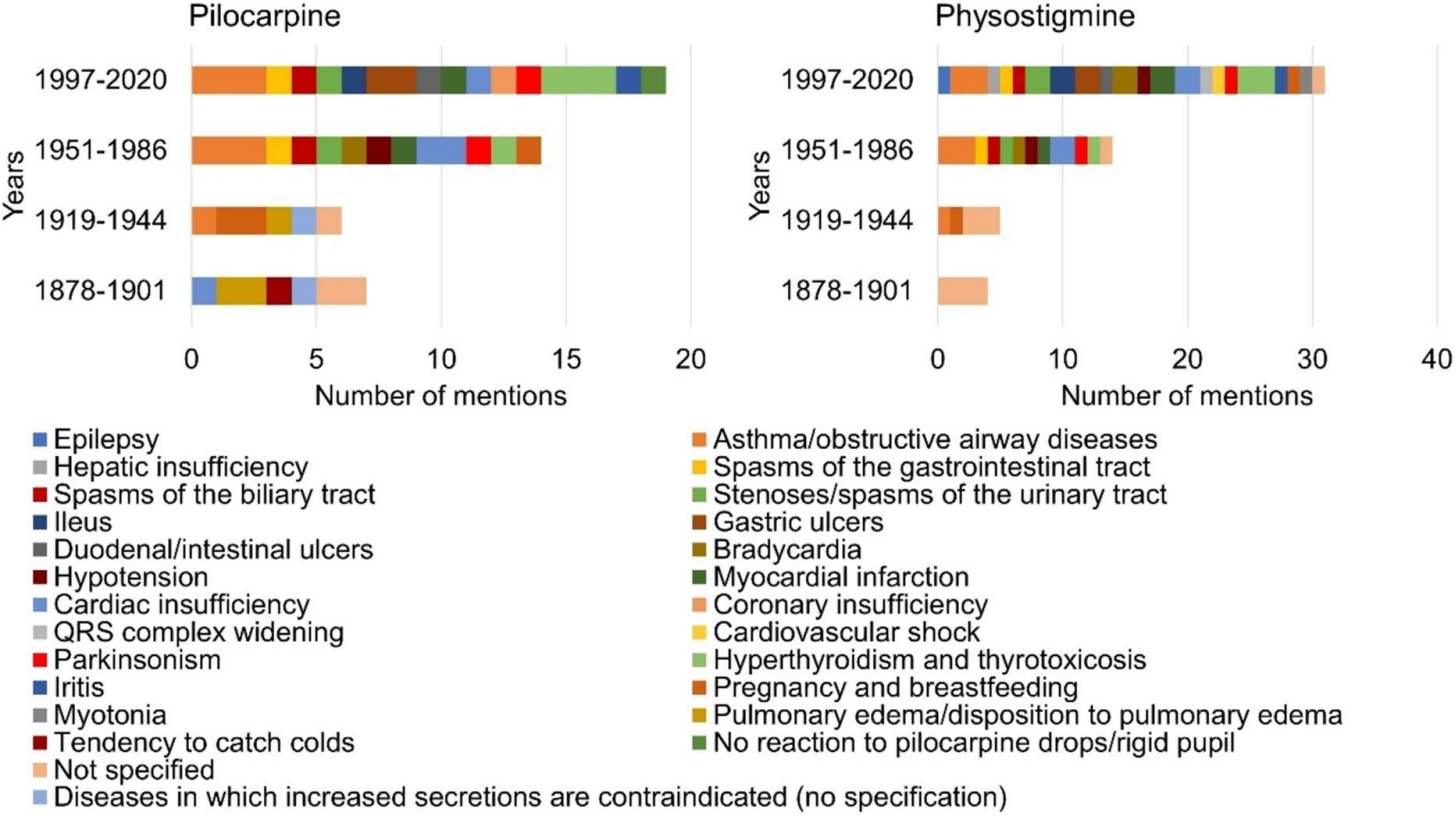
Information on contraindications of the active substances*.* The contents given for pilocarpine are shown in the left panel. The right panel shows the contraindications listed for physostigmine. The absolute amount of information is shown

Figure 12 shows the contraindications of pilocarpine and physostigmine listed by textbook group. The scope of the content presented on the contraindications of pilocarpine increased from the second to the fourth textbook group (see supplemental Table S15). The textbooks in the first and second textbook groups each listed four different contraindications for pilocarpine. The textbooks in the third textbook group listed eleven contraindications. In the fourth textbook group, the information could be assigned to fourteen different contraindications. A significant increase in the amount of information on the contraindications of physostigmine could be observed from the first to the fourth textbook group (see supplemental Table S16). In the textbooks of the first textbook group, there was no content on the contraindications of physostigmine therapy. The textbooks in the second textbook group listed two different contraindications. The textbooks in the third textbook group mentioned a total of ten different contraindications. In the fourth textbook group, information on twenty contraindications of physostigmine was given.

In total, the number of contraindications listed increased significantly for both pilocarpine and physostigmine by 2020. Overall, the number of contraindications mentioned for both pilocarpine and physostigmine is inversely proportional to the number of indications for both drugs in the period 1878–2020.

## Discussion of incorrect content

**Fig. 13:**
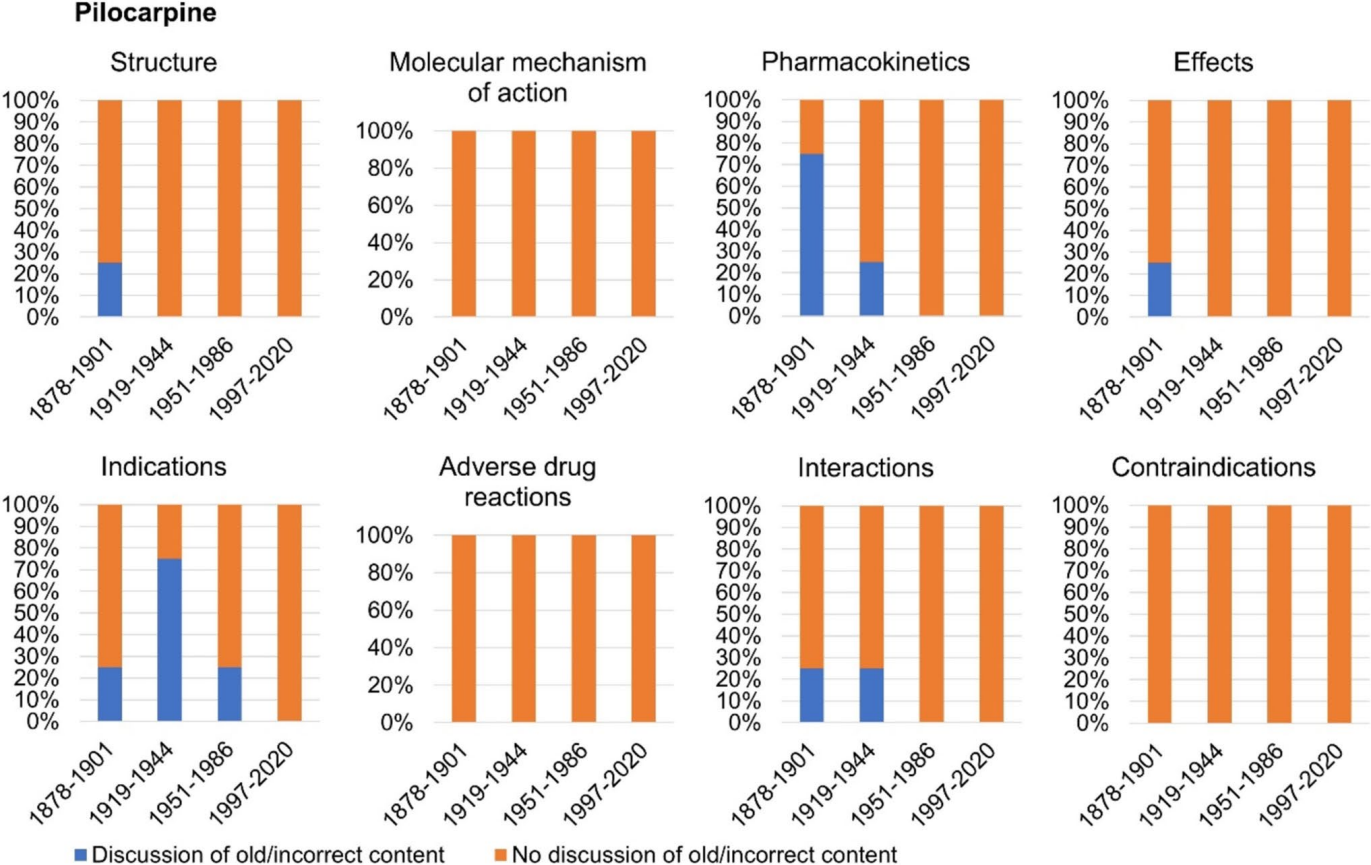
Discussion of old and incorrect content regarding pilocarpine. It was analyzed whether outdated and incorrect content is named and discussed as such in the textbooks. Only content that could be assigned to one of the eight analysis categories was included in the analysis. All content directly or indirectly related to pilocarpine was included in the analysis. The proportion of textbooks in which incorrect content is discussed is given as a percentage of the total number of textbooks per textbook group

Figure 13 shows whether outdated and incorrect content regarding pilocarpine is addressed as such in the textbooks. In the categories *molecular mechanism of action*, *adverse drug reactions*, and *contraindications*, 100% of the textbooks from the period 1878–2020 did not discuss any old or incorrect content (see Fig. 13). Incorrect contents of the categories *structure* and *effects* were discussed by 25% of the textbooks of the first textbook group. Twenty-five percent of the textbooks in the first textbook group and second textbook group discussed old content in the category *interactions*. Outdated content in the category *pharmacokinetics* was addressed by 75% of the textbooks in the first textbook group and 25% of the textbooks in the second textbook group. Incorrect content in the category *indications* was discussed the most over the entire study period from 1878 to 2020. Twenty-five percent of the textbooks in the first textbook group, 75% of the textbooks in the second textbook group, and 25% of the textbooks in the third textbook group addressed outdated knowledge regarding the indications of pilocarpine. Modern textbooks of the fourth textbook group did not address any old and incorrect content.

**Fig. 14:**
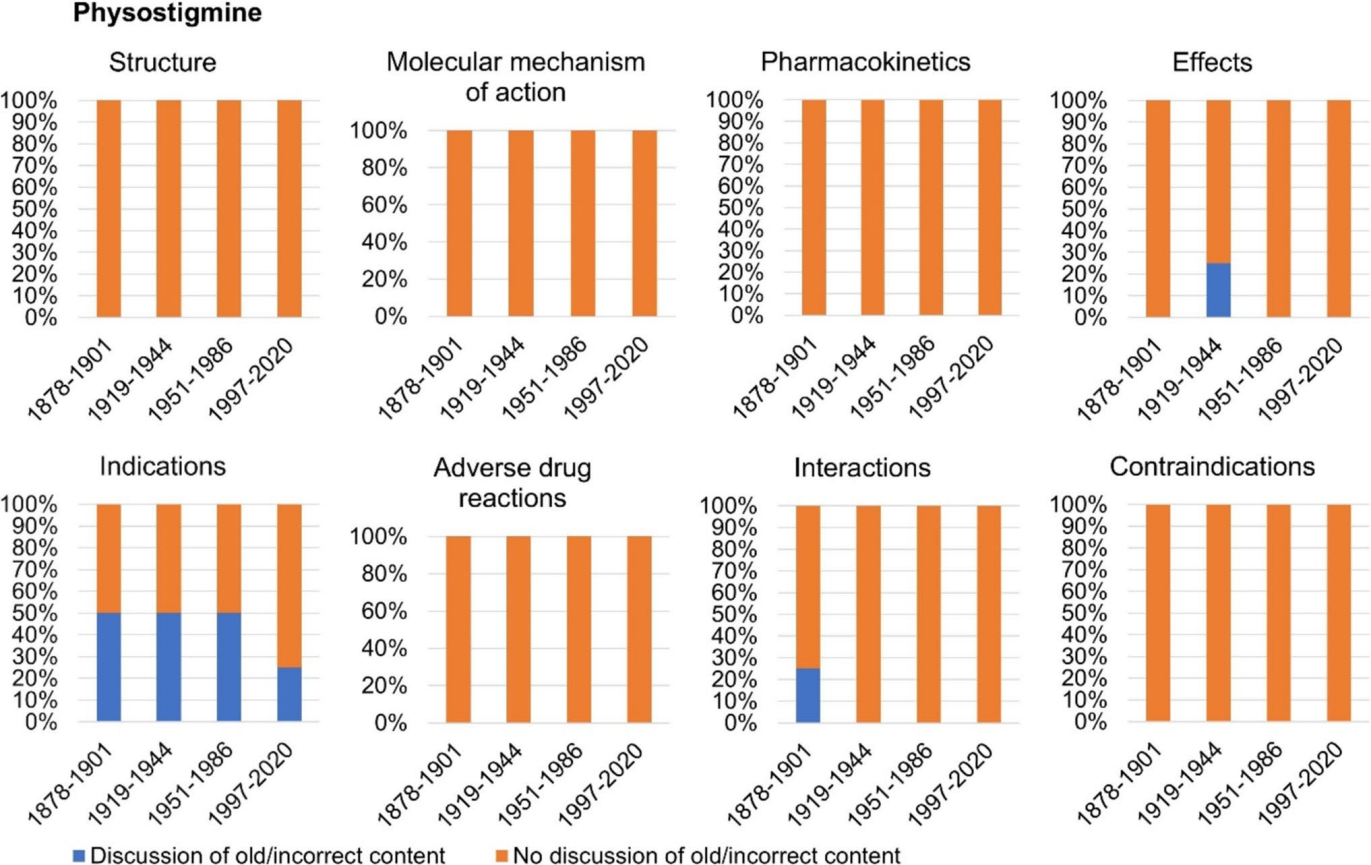
Discussion of old and incorrect content regarding physostigmine. It was analyzed whether outdated and incorrect content is named and discussed as such in the textbooks. Only content that could be assigned to one of the eight analysis categories was included in the analysis. All content directly or indirectly related to pilocarpine or physostigmine was included in the analysis. The proportion of textbooks in which incorrect content is discussed is given as a percentage of the total number of textbooks per textbook group

Figure 14 illustrates whether outdated and incorrect content is addressed as such in the textbooks regarding physostigmine. One hundred percent of the textbooks from the period 1878–2020 did not discuss any old or incorrect content in the categories *structure*, *molecular mechanism of action*, *pharmacokinetics*, *adverse drug reactions*, and *contraindications* (see Fig. 14)*.* Incorrect contents of the category *interactions* were discussed by 25% of the textbooks of the first textbook group. Twenty-five percent of the textbooks in the second textbook group discussed old content in the category *effects*. Incorrect content in the category *indications* was addressed the most, as 50% of the textbooks in the first, second, and third textbook group and 25% of the textbooks in the fourth textbook group discussed outdated knowledge. Thus, the category *indications* is the only category in which outdated knowledge is discussed by modern textbooks from 1997 onwards.

Table 2 shows which indications were named as outdated and incorrect in the textbooks. There were only similarities between textbook 2 and textbook 6 as well as textbook 8 and textbook 9 regarding the outdated indications covered (see Table 2). This is most likely because textbooks 2 and 6 were both written by Oswald Schmiedeberg and textbooks 8 and 9 also have the same author, Fritz Eichholtz. This suggests that it depends heavily on the author which outdated indications are discussed in the textbooks.

**Table 2:** Outdated and incorrect indications of physostigmine mentioned in the textbooks. Only those textbooks are listed which contained information on outdated and incorrect indications

Discussed old/incorrect indications							
Years	Textbooks	Diseases of the CNS	Tetanus	Tachycardia	Intestinal excitant	Myasthenia gravis	Intoxication with tricyclic antidepressants
1883	2	x					
1901	4		x				
1921	6	x					
1944	8			x	x		
1951	9			x			
1977	11					x	
2016	15						x

All in all, outdated and incorrect information on the structure and pharmacokinetics was discussed exclusively for pilocarpine, whereas modern textbooks from 1997 onwards only addressed incorrect and old physostigmine indications. This shows pronounced differences between pilocarpine and physostigmine in the discussion of outdated content. Overall, mainly older books address outdated content, with the category *indications* for physostigmine being an exception. Nonetheless, the outdated content addressed—as in the example of physostigmine indications—depends on the author. Accordingly, older textbooks are better at discussing advances in scientific concepts than newer textbooks. Newer textbooks tend to simply state current knowledge without discussing the dynamics of knowledge development. Thereby, modern textbooks mostly miss a great opportunity to educate students about the history of science which is a history of change of concepts and facts.

## Drug prescriptions

**Fig. 15:**
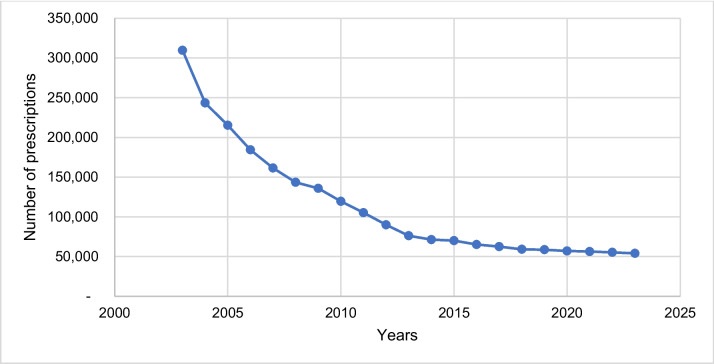
The number of drug prescriptions for pilocarpine in the period 2003–2023 in Germany is shown. The number of prescriptions of preparations with ATC codes N07AX01 and S01EB01 are shown in total. From 2016 onwards, formulations with pilocarpine are also included in the prescription figures. The data is based on the prescription figures of the Scientific Institute of the AOK (WidO)

Figure 15 shows the number of drug prescriptions for pilocarpine in the period 2003–2023 in Germany. The maximum number of pilocarpine prescriptions was found in 2003 with 309,637 prescriptions (see Fig. 15). Over the course of the 2003–2023 study period, the number of prescriptions fell exponentially and reached its minimum in 2023 with 54,078 prescriptions.

**Fig. 16:**
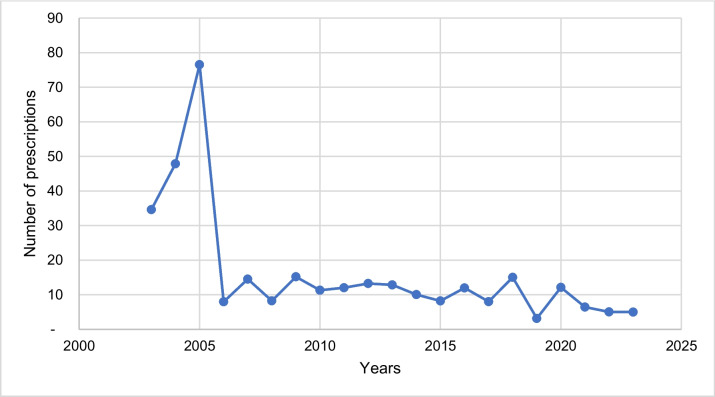
The number of drug prescriptions for physostigmine in the period 2003–2023 in Germany is shown. The number of prescriptions of preparations with ATC code V03AB19 is shown. The data is based on the prescription figures of the Scientific Institute of the AOK (WIdO)

Figure 16 shows the number of drug prescriptions for physostigmine in the period 2003–2023. In Germany, the number of physostigmine prescriptions reached its maximum in 2005 with just 77 prescriptions (see Fig. 16). Subsequently, the number of prescriptions dropped significantly to just 8 in 2006. In 2019, physostigmine was prescribed the least with a total of 3 prescriptions. In 2023, the number of prescriptions was 5. Thus, clinically, physostigmine is virtually non-existent.

When comparing the prescription figures for physostigmine with those for reserpine from the study by Misera and Seifert (2023), it was noticeable that the obsolete drug reserpine, with 70,500 prescriptions in 2016, still had significantly more prescriptions than physostigmine with only 12 prescriptions in the same year. It should also be noted that most current textbooks no longer listed an indication for reserpine, whereas all newer textbooks in the fourth textbook group (textbooks 13, 14, 15, and 16) continued to list indications for physostigmine (Misera and Seifert 2023). This shows a striking discrepancy between the content depicted in textbooks and the actual clinical practice and prescription reality of the drug. Therefore, in the case of physostigmine, pharmacological textbooks are very far lagging clinical practice.

Overall, the number of prescriptions for both drugs fell significantly between 2003 and 2023, meaning that their therapeutical relevance and use in the treatment of diseases is reduced today. However, this trend is much more pronounced for physostigmine, which is why the importance and benefit of physostigmine as a drug is now exceedingly limited. Accordingly, the drug can be considered obsolete today. It can also be seen that the therapeutic relevance of pilocarpine—based on the number of prescriptions since 2003—was always much higher than the one of physostigmine.

## PubMed and Google Scholar search results

**Fig. 17:**
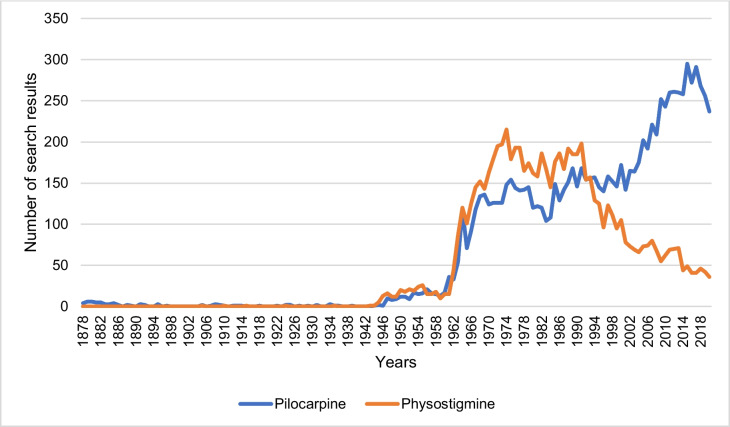
Number of PubMed search results for pilocarpine and physostigmine. The number of pilocarpine and physostigmine entries per year for the study period 1878–2020 is shown. No filters were applied to the search in terms of text availability, article attribute, article type, or language

Figure 17 shows the annual number of PubMed search results for pilocarpine and physostigmine for the study period 1878–2020. In the period 1878–1960, the number of PubMed search results for pilocarpine and physostigmine was at a low level with 0–21 entries per year for pilocarpine and 0–26 entries for physostigmine (see Fig. 17). Subsequently, the number of search results for both drugs increased, so the number of entries for physostigmine reached its maximum in 1973 with 197 PubMed results. Overall, there were no significant differences in the quantitative development of the PubMed search results for both drugs in the period 1878–1993. From 1994 onwards, the number of PubMed entries for pilocarpine continued to rise and reached its maximum in 2015 with 295 PubMed entries. In 2020, there were 237 search results for pilocarpine in PubMed. In contrast, the number of physostigmine entries decreased from 1994 onwards. In 2020, there were a total of 36 search results for physostigmine. Therefore, there were significantly more PubMed search entries for pilocarpine than for physostigmine in 2020. But compared to clinical practice, physostigmine is still quite prominent in Pubmed, potentially conveying the impression of still existing clinical relevance.

Google Scholar is quite popular among researchers, but the database is controversial because of the potential inclusion of predatory content. But for the sake of completeness, search results in Google Scholar on pilocarpine and physostigmine for the period 1878–2020 were also analyzed (see supplemental Fig. S12). In Google Scholar, the number of hits for pilocarpine increased exponentially over time, in sharp contrast to prescription numbers. For physostigmine, an almost constant number of hits over the past 30 years was noted which does not reflect at all clinical reality. Thus, looking at the number of Google Scholar hits can be quite misleading as an indicator of clinical relevance of a drug. This result came as a surprise to us. It was also quite surprising that Pubmed and Google Scholar yield so diverging results on pilocarpine and physostigmine, calling for caution to use these two databases interchangeably.

## Comparison of German textbooks with the US pharmacology textbook “Goodman & Gilman’s”

Table 3 shows the results of the analysis of four editions of *Goodman & Gilman’s: The Pharmacological Basis of Therapeutics* from 1996, 2006, 2018, and 2023 to compare them with the German textbooks in the fourth textbook group from 1997 to 2020. In the most recent textbooks from 2020 (Germany) and 2023 (USA), the relative proportion of active substance pages in the total number of pages of the textbook was greater for both pilocarpine (0.30%) and physostigmine (0.43%) in the US textbooks than in the German textbooks (pilocarpine: 0.28%, physostigmine: 0.19%) (see Table 3 and Fig. 2). Especially for physostigmine, there was a marked difference between the scope of the drug in US and German textbooks, suggesting a potentially different clinical relevance and use of the drug in the US and Germany. Overall, pilocarpine and physostigmine are currently considered more clinically relevant in the US than in Germany.

**Table 3:** Exemplary analysis of four editions of *Goodman & Gilman’s: The Pharmacological Basis of Therapeutics* from 1996, 2006, 2018, and 2023. The textbooks Hardman et al. (1996), Brunton et al. (2006), Brunton et al. (2018) and Brunton and Knollmann (2023) were analyzed. The editions were selected based on the publication years of the German textbooks in the fourth textbook group. The results were compared to the German textbooks in the fourth textbook group. The textbooks were evaluated regarding the scope of the drugs in the textbooks, the indications given, and the discussion of outdated content

Years	Scope in the textbooks		Indications		Discussion of incorrect content	
	Pilocarpine	Physostigmine	Pilocarpine	Physostigmine	Pilocarpine	Physostigmine
1996	Relative proportion in the total number of pages: 0.42%	Relative proportion in the total number of pages: 0.63%	Ophthalmologic diseases, metabolic and autoimmune diseases, and others were mentioned; focus on ophthalmologic diseases	Ophthalmologic diseases, diseases of the nervous and skeletal system, intoxications, and others were mentioned; focus on ophthalmologic diseases	No discussion of outdated and incorrect content	Discussion of outdated and incorrect indications
2006	Relative proportion in the total number of pages: 0.49%	Relative proportion in the total number of pages: 0.45%	Ophthalmologic diseases, metabolic and autoimmune diseases, and others were mentioned; focus on ophthalmologic diseases	Ophthalmologic diseases, diseases of the nervous and skeletal system, intoxications, and others were mentioned; focus on ophthalmologic diseases	No discussion of outdated and incorrect content	Discussion of outdated and incorrect indications
2018	Relative proportion in the total number of pages: 0.56%	Relative proportion in the total number of pages: 0.42%	Ophthalmologic diseases, metabolic and autoimmune diseases, and others were mentioned; focus on ophthalmologic diseases	Ophthalmologic diseases, diseases of the nervous system and skeletal muscle system, and intoxications were mentioned; no content focus could be identified	Discussion of outdated and incorrect indications	No discussion of outdated and incorrect content
2023	Relative proportion in the total number of pages: 0.30%	Relative proportion in the total number of pages: 0.43%	Ophthalmologic diseases, metabolic and autoimmune diseases, and others were mentioned; focus on ophthalmologic diseases	Ophthalmologic diseases, diseases of the nervous and skeletal muscle system, and intoxications were mentioned; no content focus could be identified	Discussion of outdated and incorrect indications	No discussion of outdated and incorrect content

Regarding the listed indications in the US textbooks, for pilocarpine, ophthalmological, metabolic, and autoimmune diseases as well as the increase of saliva secretion were listed in the textbooks from 1996 to 2023 (see Table 3). The indications for pilocarpine in German textbooks are rather similar, but gastrointestinal indications of pilocarpine were only mentioned here (see Fig. 8). For physostigmine, ophthalmological, nervous system, and skeletal muscle diseases, intoxications and infestation of the eyelashes by lice and mites were mentioned as indications in the US textbooks (see Table 3). In German textbooks in the fourth textbook group, only ophthalmological diseases and intoxications were mentioned as indications of physostigmine (see Fig. 8). Thus, differences between the described indications of both drugs between German and US-American textbooks were found with physostigmine being therapeutically more relevant in the US than in Germany.

Fifty percent of the US textbooks addressed outdated or incorrect content for both drugs in the category *indications* (see Table 3). Outdated content in other categories was not discussed. This underlines the previously described results of German-language textbooks and shows that both modern German and US pharmacology textbooks have significant deficits in discussing outdated or incorrect content and therefore portraying the change of pharmacological knowledge (see Figs. 13 and 14).

## Comparison between pilocarpine, physostigmine, and hydrogen cyanide

Table 4 shows a comparison of study results for pilocarpine, physostigmine, and hydrogen cyanide. In the categories *scope in the textbooks*, *structure*, *molecular mechanism of action*, *pharmacokinetics*, *effects*, *indications*, *interactions*, and *contraindications*, pilocarpine and physostigmine showed similar developments in terms of time and content (see Table 4). Accordingly, there was a change in the content focus for both drugs in the categories *structure* and *molecular mechanism of action* from 1951 onwards. In the categories *interactions* and *contraindications*, there was an increase in the number of interactions and contraindications mentioned for both pilocarpine and physostigmine by 2020. The greatest differences between pilocarpine and physostigmine could be found in the category *adverse drug reactions*, as the scope of side effects presented for pilocarpine was reduced from 1919 onwards and increased for physostigmine at the same time. Overall, the changes in the presentation of pilocarpine and physostigmine in the textbooks show pronounced similarities.

**Table 4:** Comparison of study results for pilocarpine, physostigmine, and hydrogen cyanide. The data on hydrogen cyanide were taken from the publication by Ludwig and Seifert (2024). The most pronounced changes regarding the scope and given content of a category are shown for all categories analyzed. Regarding the analysis of the scope of drug-related content in the textbooks, the time of the maximum and minimum relative share of the active substance pages of the total number of pages is listed for comparison

Categories	Pilocarpine	Physostigmine	Hydrogen cyanide
Scope in the textbooks	Maximum: 1933 (3.67%), Minimum: 1997 (0.13%)	Maximum: 1933 (2.86%), Minimum: 2020 (0.19%)	Maximum: 1933 (2.86%), Minimum: 1997 (0.26%)
Structure	From 1951 onwards: focus on structural formula	From 1951 onwards: focus on structural formula	Constant focus on sum formula
Molecular mechanism of action	From 1951 onwards: focus on activation of muscarinic acetylcholine receptors	From 1951 onwards: focus on reversible/substrate inhibition of acetylcholinesterase	From 1951 onwards: focus on binding to Fe^3+^ of cytochrome oxidase
Pharmacokinetics	From 1997 onwards: decreased focus on the drug’s application and onset and duration of action and more content on the elimination, absorption, and metabolism	From 1997 onwards: decreased focus on the drug’s application and onset and duration of action and more content on the distribution, elimination, and metabolism	Not analyzed
Effects	Constant focus on peripheral secretion processes, increased scope from 1919 onwards	From 1951 onwards: focus on effects on peripheral secretions, increased scope from 1919 onwards	Decreased scope from 1919 onwards
Indications/Areas of application	Constant focus on ophthalmological diseases, number of indications decreased from 1878 onwards	From 1997 onwards: focus on treatment of intoxications, number of indications decreased from 1878 onwards	Number of areas of application decreased from 1878 onwards
Adverse drug reactions	Number of ADRs decreased from 1878 onwards	Number of ADRs increased from 1919 onwards	Not analyzed
Interactions	From 1997 onwards: focus on interactions with parasympatholytics and beta-receptor antagonists, number of indications increased from 1878 onwards	From 1997 onwards: focus on interactions with atropine, curare, and other non-depolarizing muscle relaxants and beta-receptor antagonists, number of indications increased from 1878 onwards	Not analyzed
Contraindications	Number of contraindications increased from 1919 onwards	Number of contraindications increased from 1878 onwards	Not analyzed

A further comparison of pilocarpine and physostigmine with hydrogen cyanide showed similar developments for all three substances in the categories *scope in the textbooks*, *molecular mechanism of action*, and *indications* respectively *areas of application* for hydrogen cyanide. The relative proportion of substance-related pages in the total number of pages in the textbooks was at its maximum in 1933 (textbook 7) for pilocarpine, physostigmine, and hydrogen cyanide. The minimum relative share of substance-related pages was also found for all drugs in the period 1997–2020 and thus in textbooks of the fourth textbook group. In the category *molecular mechanism of action*, a change in content focus could be seen for all analyzed substances from 1951 onwards. In the category *indications* respectively *areas of application*, the number of indications and applications mentioned decreased from 1878 onwards for all drugs. In contrast, in the categories *structure* and *effects*, clear differences could be seen between pilocarpine/physostigmine and hydrogen cyanide. This points to drug-specific non-linearities in pharmacological knowledge development as the differences between pilocarpine/physostigmine and hydrogen cyanide are more pronounced compared to the differences between only pilocarpine and physostigmine.

## Limitations

Due to the restriction of the analysis to German-language textbooks and just one English-language textbook from the US, the transfer of the results to the entire international literature is not guaranteed. Specifically, pharmacology textbooks from the UK, Japan, China, and India should be examined as well. Furthermore, we analyzed just one textbook per decade. The information on pilocarpine and physostigmine in the textbooks by Schmiedeberg 1883 and 1921 (textbooks 2 and 6), as well as by Eichholtz 1944 and 1951, (textbooks 8 and 9) is similar. Since the key word index was used as the basis for the analysis and selection of the analyzed pages, only the information on the drugs listed on the pages of the key word index was included in the analysis.

## Conclusions and future studies

Pharmacology textbooks are used by students and doctors as a learning and reference tool, which is why they are of great importance for medical practice. Within the case study of pilocarpine and physostigmine, we showed that the pharmacological knowledge on the molecular mechanism, chemical structure, and pharmacokinetics of both drugs changed the most during the period of 150 years. The textbooks did not mention a molecular mechanism of action of pilocarpine until 1944 and only described the activation of muscarinic acetylcholine receptors as the molecular basis of pilocarpine’s effect from 1951 onwards. Until 1944, most textbooks on physostigmine also did not mention the molecular mechanism of action. From 1951 onwards, the reversible inhibition of acetylcholinesterase by physostigmine is mentioned as the mechanism of action. In the categories *effects*, *indications*, *adverse drug reactions*, *interactions*, and *contraindications*, the detected changes in the pharmacological knowledge presented were smaller compared to the categories *molecular mechanism of action*, *structure*, and *pharmacokinetics*.

When comparing the two drugs, there were similar changes in terms of scope or content focus in the categories *structure*, *molecular mechanism of action*, *pharmacokinetics*, *effects*, *indications*, *interactions*, and *contraindications*. Only in the category *adverse drug reactions* did pilocarpine and physostigmine differ regarding the scope of the given content. Overall, this shows a similar development of pharmacological knowledge regarding pilocarpine and physostigmine during the study period. In contrast, the comparison of the two drugs with hydrogen cyanide showed more differences, pointing to drug-specific non-linearities in knowledge development (Ludwig and Seifert 2024).

A decrease in the representation of pilocarpine and physostigmine in pharmacological textbooks, based on the relative share of pages with content on pilocarpine and physostigmine in the total number of pages of the textbook, was observed during the study period. But if the textbook presentation of the two drugs is placed into context with clinical relevance, pilocarpine, and physostigmine are substantially over-represented in modern textbooks, potentially transmitting the impression of substantial clinical relevance of the two drugs to unexperienced students. Apparently, the presentation of a drug in previous textbooks has a substantial impact (burden) on how the drug is presented in a new textbook. This historic burden is particularly evident for physostigmine. The drug still survives in modern textbooks without proper reference to its clinical obsolescence for decades. Such presentations should be avoided in textbooks because students, looking at prescription numbers of a drug as an indicator for clinical relevance, may come to the incorrect conclusion that pharmacology textbooks in general are outdated and need not to be read anymore, turning to allegedly more “modern” teaching materials.

Therefore, it is important that future pharmacology textbooks focus less on the content of previous textbooks and instead base the scope and content on a drug on current research data and prescription figures to best reflect the actual clinical relevance and use of drugs. In the case of pilocarpine and physostigmine, this would mean to reduce the scope of the drugs in the textbooks and adapting the areas of application presented to the actual reality of prescribing and current research data. In addition, new textbooks should also clarify outdated and incorrect knowledge about obsolete drugs and the reasons for their reduced clinical relevance today. The presentation of other drugs that have become obsolete due to their kinetics, an unfavorable balance between desired and undesired effects, or the availability of more effective drugs should also be adapted in future textbooks in the manner described.

Based on the present study and the studies by Misera and Seifert (2023) as well as Ludwig and Seifert (2024) it will be worthwhile to analyze the presentation of other drugs and poisons in pharmacology textbooks. Such analysis must explicitly include the many English-language textbooks that have a far greater international reach than German-language textbooks, especially since we have already identified differences in the presentation of the drugs in US and German textbooks.

Pharmacological knowledge develops non-linearly for different aspects of a given drug, and it cannot be taken for granted that a current textbook provides up-to-date information. The case study of pilocarpine and physostigmine shows that pharmacology has a long history of errors with respect to mechanism of action, chemical structure, and pharmacokinetics that were ultimately corrected. But since the history of each drug is unique, this type of analysis should be extended to other drugs to decipher general mechanisms and historic triggers underlying the development of pharmacological knowledge.

A somewhat unexpected avenue of future research emerging form this case study concerns the analysis of scientific databases. It will have to be analyzed in depth for which reason Pubmed and Google Scholar yielded quite different search results, particularly for physostigmine. This result contradicts the general assumption and common wisdom of scientists that both databases give consistent results with Google Scholar resulting in higher number of hits (potentially due to predatory content).

## Nine simple take-home messages for (pharmacology) textbook authors

1. **The importance of prescription numbers:** Pharmacology textbook authors should carefully check current drug prescription numbers before deciding about the inclusion or non-inclusion of a drug into a textbook.
2. **e-Function as indicator for obsolescence:** The decline of clinical relevance, as a rule, follows an exponential function, like the decay of a radioactive isotope. Thus, the exponential decline in prescription numbers is a robust indicator of developing obsolescence. Drugs with such exponential decline should be flagged accordingly. The exponential decline of prescription numbers can be easily detected already in the initial phase. Thus, textbooks can be proactive at anticipating future developments. The anticipation of the future fate of a drug has not yet been integrated in any pharmacology textbook.
3. **Start with an empty screen:** Consulting previous editions of a textbook or other related textbook will likely result in the recapitulation of clinically irrelevant content and impede with the generation of future-oriented content. Therefore, starting a textbook without consulting previous textbooks is a good idea.
4. **Don’t trust hit numbers:** Consulting Pubmed or particularly Google Scholar for the clinical relevance of a topic based on the number of hits can be quite misleading. Caution should be exerted when analyzing hit numbers.
5. **Foster the skill of critical thinking:** Pharmacology textbooks should not be simply regarded as “students’ digest” formats of current pharmacological knowledge. Rather, modern textbooks should openly discuss historic developments and errors as a source to foster the skill of “critical thinking.” Older textbooks, in general, did a better job in this regard than the newer ones. The ability to think critically is crucial for our students to make prudent medication decisions in the future and to discriminate between true facts and fake facts, circulating unfiltered on the internet.
6. **History as the basis of the future:** It is acceptable to include obsolete drugs into modern textbooks if the textbook authors explicitly explain why the respective drug has fallen into obsolescence. This provides a unique opportunity for students to learn from history and that pharmacological knowledge changes constantly and will continue to do so in the future.
7. **Only pharmacology textbooks can teach concepts:** If pharmacology textbook authors take the easily implementable recommendations under points 1–6 at heart, pharmacology textbooks will have a bright future because PowerPoint slides, video tutorials, online dictionaries, and artificial intelligence-generated teaching content typically fail at integrating these aspects. Such formats are particularly poor at conveying overarching scientific concepts and paradigms.
8. **Other fields with a decline in textbook use can benefit as well:** The considerations under points 1–7 can also be applied to other fields of science where textbooks are threatened by extinction due to the emergence of other allegedly more “modern” formats of teaching.
9. **Don’t forget the cultural aspects of pharmacology:** While the molecular pharmacology of drugs has no cultural dimension, the clinical pharmacology of drugs has such a dimension. It is important to look across the fence and analyze how different countries assess and use a given drug.

## Supplementary Information

Below is the link to the electronic supplementary material.

Supplementary file1 (DOCX 3766 KB)

## Data Availability

All source data for this work are available upon reasonable request.

## List of abbreviations

ADME: Absorption, distribution, metabolism, and excretion
ADR: Adverse drug reactions
AOK: General local health insurance
ATC: Anatomical-therapeutic-chemical classification of active substances and medicinal products
CNS: Central nervous system
GKV: Statutory health insurance
mg: Milligram
R: Coefficient of determination
US: United States
USA: United States of America
WIdO: Scientific Institute of the AOK
