# Supplementary material for: The decline in the clinical relevance of pilocarpine and physostigmine monitored in pharmacology textbooks from 1878 to 2023: nine take-home messages for future (pharmacology) textbook authors
Source: Naunyn Schmiedebergs Arch Pharmacol. 2024 Nov 12;398(5):5171–93. doi: 10.1007/s00210-024-03558-x (PMC11985697; doi:10.1007/s00210-024-03558-x)
Supplement: Supplementary file 1 — Supplementary file1 (DOCX 3766 KB) [file 210_2024_3558_MOESM1_ESM.docx]

**The decline in the clinical relevance of pilocarpine and physostigmine monitored in pharmacology textbooks from 1878-2023: Nine take-home messages for future (pharmacology) textbook authors**

**Laureen Ludwig and Roland Seifert**

**Supplemental figures and tables**

| Coding | Structure | | 1878-1901 | 1919-1944 | 1951-1986 | 1997-2020 | All | Range |
| --- | --- | --- | --- | --- | --- | --- | --- | --- |
| 1 | Sum formula |  | 3 | 2 | 0 | 0 | 5 | 3 |
| 2 | Structural formula | | 1 | 0 | 3 | 3 | 7 | 3 |
| 3 | Not specified |  | 1 | 2 | 1 | 1 | 5 | 1 |
|  | Total mentions | | 4 | 2 | 3 | 3 | 12 |  |
|  | Average range | |  |  |  |  |  | 2.33 |

Table S1: Category "Structure" with encodings and results for pilocarpine. The frequency with which each encoding is represented per textbook group and the encoding´s range calculated from this is shown. In addition, the average range of encodings in this category was calculated. The average range of the category is rounded to two decimal places.

| Coding | Structure | | 1878-1901 | 1919-1944 | 1951-1986 | 1997-2020 | All | Range |
| --- | --- | --- | --- | --- | --- | --- | --- | --- |
| 1 | Sum formula |  | 3 | 2 | 0 | 0 | 5 | 3 |
| 2 | Structural formula | | 0 | 0 | 4 | 3 | 7 | 4 |
| 3 | Not specified |  | 1 | 2 | 0 | 1 | 4 | 2 |
|  | Total mentions | | 3 | 2 | 4 | 3 | 12 |  |
|  | Average range | |  |  |  |  |  | 3 |

Table S2: Category "Structure" with encodings and results for physostigmine. The frequency with which each encoding is represented per textbook group and the encoding´s range calculated from this is shown. In addition, the average range of encodings in this category was calculated.

| Coding | Molecular mechanism of action | | | | | 1878-1901 | 1919-1944 | 1951-1986 | 1997-2020 | All | Range |
| --- | --- | --- | --- | --- | --- | --- | --- | --- | --- | --- | --- |
| 1 | Activation of muscarinic acetylcholine receptors | | | |  | 0 | 0 | 2 | 4 | 6 | 4 |
| 2 | Activation of postganglionic acetylcholine receptors | | | |  | 0 | 0 | 1 | 0 | 1 | 1 |
| 3 | Not specified |  |  |  |  | 4 | 4 | 1 | 0 | 9 | 4 |
|  | Total mentions | |  |  |  | 0 | 0 | 3 | 4 | 7 |  |
|  | Average range | |  |  |  |  |  |  |  |  | 3 |

Table S3: Category "Molecular mechanism of action" with encodings and results for pilocarpine. The frequency with which each encoding is represented per textbook group and the encoding´s range calculated from this is shown. In addition, the average range of encodings in this category was calculated.

| Coding | Molecular mechanism of action | | | | | 1878-1901 | 1919-1944 | 1951-1986 | 1997-2020 | All | Range |
| --- | --- | --- | --- | --- | --- | --- | --- | --- | --- | --- | --- |
| 1 | Reversible/substrate inhibition of acetylcholinesterase | | | |  | 0 | 0 | 3 | 4 | 7 | 4 |
| 2 | Inhibition of acetylcholinesterase (no specification) | | | |  | 0 | 1 | 1 | 0 | 2 | 1 |
| 3 | Not specified |  |  |  |  | 4 | 3 | 0 | 0 | 7 | 4 |
|  | Total mentions | |  |  |  | 0 | 1 | 4 | 4 | 9 |  |
|  | Average range | |  |  |  |  |  |  |  |  | 3 |

Table S4: Category "Molecular mechanism of action" with encodings and results for physostigmine. The frequency with which each encoding is represented per textbook group and the encoding´s range calculated from this is shown. In addition, the average range of encodings in this category was calculated.

| Coding | Pharmacokinetics | | | | | 1878-1901 | 1919-1944 | 1951-1986 | 1997-2020 | All | Range |
| --- | --- | --- | --- | --- | --- | --- | --- | --- | --- | --- | --- |
|  | Application |  |  |  |  | 7 | 7 | 7 | 4 | 25 |  |
| 1 | Enteral application | |  |  |  | 3 | 2 | 1 | 0 | 6 | 3 |
| 2 | Parenteral application | |  |  |  | 3 | 3 | 2 | 0 | 8 | 3 |
| 3 | Topical application | |  |  |  | 1 | 2 | 4 | 4 | 11 | 3 |
|  | Absorption |  |  |  |  | 2 | 0 | 2 | 3 | 7 |  |
| 4 | Enteral Absorption | |  |  |  | 0 | 0 | 1 | 0 | 1 | 1 |
| 5 | Absorption via mucous membranes (no specification) | | | |  | 1 | 0 | 0 | 0 | 1 | 1 |
| 7 | Absorption via subcutaneous tissue | | |  |  | 1 | 0 | 0 | 0 | 1 | 1 |
| 8 | General information on resorption speed and resorption capacity | | | | | 0 | 0 | 1 | 3 | 4 | 3 |
|  | Metabolism |  |  |  |  | 0 | 0 | 1 | 2 | 3 |  |
| 12 | No degradation/hydrolysis by cholinesterase | | | |  | 0 | 0 | 1 | 2 | 3 | 2 |
|  | Excretion |  |  |  |  | 2 | 0 | 0 | 3 | 5 |  |
| 13 | Renal excretion | |  |  |  | 1 | 0 | 0 | 2 | 3 | 2 |
| 14 | Enteral excretion | |  |  |  | 1 | 0 | 0 | 0 | 1 | 1 |
| 16 | Information on plasma half-life/elimination half-life | | | |  | 0 | 0 | 0 | 1 | 1 | 1 |
|  | Onset and duration of action | | |  |  | 6 | 3 | 5 | 0 | 14 |  |
| 17 | Information on the onset of action | | |  |  | 3 | 1 | 2 | 0 | 6 | 3 |
| 18 | Information on the duration of action | | |  |  | 3 | 2 | 3 | 0 | 8 | 3 |
| 19 | Not specified |  |  |  |  | 0 | 1 | 0 | 0 | 1 | 1 |
|  | Total mentions | |  |  |  | 17 | 10 | 15 | 12 | 54 |  |
|  | Average range | |  |  |  |  |  |  |  |  | 2.00 |

Table S5: Category "Pharmacokinetics" with encodings and results for pilocarpine. The frequency with which each encoding is represented per textbook group and the encoding´s range calculated from this is shown. In addition, the average range of encodings in this category was calculated.

| Coding | Pharmacokinetics | | | | | 1878-1901 | 1919-1944 | 1951-1986 | 1997-2020 | All | Range |
| --- | --- | --- | --- | --- | --- | --- | --- | --- | --- | --- | --- |
|  | Application |  |  |  |  | 6 | 7 | 6 | 5 | 24 |  |
| 1 | Enteral application | |  |  |  | 3 | 1 | 0 | 0 | 4 | 3 |
| 2 | Parenteral application | |  |  |  | 0 | 3 | 2 | 4 | 9 | 4 |
| 3 | Topical application | |  |  |  | 3 | 3 | 4 | 1 | 11 | 3 |
|  | Absorption |  |  |  |  | 4 | 0 | 0 | 3 | 7 |  |
| 4 | Enteral Absorption | |  |  |  | 1 | 0 | 0 | 2 | 3 | 2 |
| 5 | Absorption via mucous membranes (no specification) | | | |  | 1 | 0 | 0 | 0 | 1 | 1 |
| 6 | Absorption via serous membranes | | |  |  | 1 | 0 | 0 | 0 | 1 | 1 |
| 7 | Absorption via subcutaneous tissue | | |  |  | 1 | 0 | 0 | 0 | 1 | 1 |
| 9 | Information on bioavailability | | |  |  | 0 | 0 | 0 | 1 | 1 | 1 |
|  | Distribution |  |  |  |  | 2 | 0 | 2 | 4 | 8 |  |
| 10 | Penetration of the blood-brain barrier/CNS-Permeability | | | | | 0 | 0 | 2 | 4 | 6 | 4 |
| 20 | Transfer of the active substance into the bile | | | |  | 1 | 0 | 0 | 0 | 1 | 1 |
| 21 | Transfer of the active substance into the saliva | | | |  | 1 | 0 | 0 | 0 | 1 | 1 |
|  | Metabolism |  |  |  |  | 0 | 0 | 1 | 4 | 5 |  |
| 11 | Degradation/hydrolysis by cholinesterase | | | |  | 0 | 0 | 1 | 4 | 5 | 4 |
|  | Excretion |  |  |  |  | 1 | 0 | 0 | 2 | 3 |  |
| 13 | Renal excretion | |  |  |  | 1 | 0 | 0 | 1 | 2 | 1 |
| 15 | Information on half-life | |  |  |  | 0 | 0 | 0 | 1 | 1 | 1 |
|  | Onset and duration of action | | |  |  | 5 | 6 | 5 | 1 | 17 |  |
| 17 | Information on the onset of action | | |  |  | 3 | 3 | 2 | 0 | 8 | 3 |
| 18 | Information on the duration of action | | |  |  | 2 | 3 | 3 | 1 | 9 | 2 |
| 19 | Not specified |  |  |  |  | 1 | 0 | 0 | 0 | 1 | 1 |
|  | Total mentions | |  |  |  | 18 | 13 | 14 | 19 | 64 |  |
|  | Average range | |  |  |  |  |  |  |  |  | 2.00 |

Table S6: Category "Pharmacokinetics" with encodings and results for physostigmine. The frequency with which each encoding is represented per textbook group and the encoding´s range calculated from this is shown. In addition, the average range of encodings in this category was calculated.

| Coding | Effects |  |  |  |  | 1878-1901 | 1919-1944 | 1951-1986 | 1997-2020 | All | Range |
| --- | --- | --- | --- | --- | --- | --- | --- | --- | --- | --- | --- |
|  | Cardiovascular system | |  |  |  | 12 | 11 | 8 | 8 | 39 |  |
| 1 | Negative chronotropy, inotropy or dromotropy | | | |  | 2 | 3 | 3 | 4 | 12 | 2 |
| 2 | Positive chronotropy, inotropy or dromotropy | | | |  | 3 | 1 | 1 | 0 | 5 | 3 |
| 3 | Vasodilatation | |  |  |  | 4 | 3 | 4 | 4 | 15 | 1 |
| 11 | Inhibition of the cardiac inhibitory fibers of the vagus nerve | | | | | 1 | 1 | 0 | 0 | 2 | 1 |
| 12 | Excitation of the cardiac inhibitory fibers of the vagus nerve | | | | | 2 | 3 | 0 | 0 | 5 | 3 |
|  | Eyes |  |  |  |  | 5 | 14 | 10 | 10 | 39 |  |
| 4 | Contraction of the sphincter pupillae muscle/miosis | | | |  | 4 | 4 | 4 | 4 | 16 | 0 |
| 5 | Contraction of the ciliary muscle | | |  |  | 0 | 2 | 3 | 4 | 9 | 4 |
| 6 | Contraction of the smooth muscles of the eye (no specification) | | | | | 0 | 1 | 0 | 0 | 1 | 1 |
| 7 | Paralysis of the oculomotor nerve | | |  |  | 0 | 1 | 0 | 0 | 1 | 1 |
| 8 | Reduction of the intraocular pressure | | |  |  | 0 | 4 | 2 | 2 | 8 | 4 |
| 9 | Increase of the intraocular pressure | | |  |  | 0 | 1 | 0 | 0 | 1 | 1 |
| 10 | Inhibition of the aqueous humor formation | | | |  | 0 | 0 | 1 | 0 | 1 | 1 |
| 49 | Excitation of the oculomotor nerve | | |  |  | 1 | 1 | 0 | 0 | 2 | 1 |
|  | Peripheral secretory processes | | |  |  | 23 | 17 | 19 | 23 | 82 |  |
| 23 | Increase in tear secretion/stimulation of the tear secretion nerves | | | | | 2 | 1 | 0 | 3 | 6 | 3 |
| 24 | Increase in saliva secretion/stimulation of the salivary secretory nerves | | | | | 4 | 4 | 4 | 4 | 16 | 0 |
| 25 | Increase in bronchial secretion, contraction and ciliated epithelial movements | | | | | 3 | 4 | 4 | 4 | 15 | 1 |
| 26 | Increase in sweat secretion/stimulation of the sweat secretion nerves | | | | | 4 | 4 | 4 | 4 | 16 | 0 |
| 27 | Increase in nasal secretion | |  |  |  | 1 | 0 | 0 | 0 | 1 | 1 |
| 28 | Increase in tracheal secretion and contraction | | | |  | 1 | 0 | 1 | 0 | 2 | 1 |
| 29 | Increase in cerumen secretion | | |  |  | 1 | 0 | 0 | 0 | 1 | 1 |
| 30 | Increase in milk secretion in breastfeeding women | | | |  | 1 | 0 | 0 | 0 | 1 | 1 |
| 33 | Inhibition of secretions (no specification) | | |  |  | 1 | 0 | 0 | 0 | 1 | 1 |
| 34 | Changes in saliva consistency and composition | | | |  | 1 | 0 | 0 | 0 | 1 | 1 |
| 35 | Changes in urine composition | | |  |  | 1 | 0 | 0 | 0 | 1 | 1 |
| 42 | Increased insulin secretion in the pancreas | | | |  | 0 | 0 | 0 | 2 | 2 | 2 |
| 43 | Increased adrenaline release in the adrenal medulla | | | |  | 0 | 0 | 1 | 1 | 2 | 1 |
| 44 | Increased histamine release in the mast cells | | | |  | 0 | 0 | 0 | 1 | 1 | 1 |
| 50 | Increase in mucus secretion | | |  |  | 0 | 1 | 1 | 0 | 2 | 1 |
|  | Sexual organs | |  |  |  | 3 | 3 | 3 | 1 | 10 |  |
| 36 | Contraction/variable effect on the uterus | | |  |  | 2 | 3 | 2 | 1 | 8 | 2 |
| 46 | Erection of the male sexual organs | | |  |  | 0 | 0 | 1 | 0 | 1 | 1 |
| 47 | Excitation of the genitals (no specification) | | | |  | 1 | 0 | 0 | 0 | 1 | 1 |
|  | Internal organs | |  |  |  | 1 | 0 | 10 | 12 | 23 |  |
| 37 | Relaxation of the sphincters of the GI tract | | | |  | 0 | 0 | 1 | 2 | 3 | 2 |
| 38 | Contraction of the bladder and detrusor vesicae muscle and increase in ureteral motility | | | | | 1 | 0 | 4 | 4 | 9 | 4 |
| 39 | Relaxation of the bladder neck muscles | | |  |  | 0 | 0 | 1 | 2 | 3 | 2 |
| 40 | Contraction/secretion of the gallbladder and sphincter of Oddi | | | | | 0 | 0 | 2 | 4 | 6 | 4 |
| 45 | Increase in glycogen synthesis in the liver | | | |  | 0 | 0 | 1 | 0 | 1 | 1 |
| 51 | Relaxation of the gallbladder sphincters | | |  |  | 0 | 0 | 1 | 0 | 1 | 1 |
| 31 | Increase in peristalsis, contraction and secretion in the gastrointestinal tract | | | | | 3 | 3 | 4 | 4 | 14 | 1 |
|  | CNS and PNS | |  |  |  | 7 | 3 | 3 | 11 | 24 |  |
| 13 | Inhibition of the vasomotor center | | |  |  | 2 | 1 | 0 | 0 | 3 | 2 |
| 14 | Activation of the respiratory center/increased breathing | | | |  | 1 | 0 | 1 | 0 | 2 | 1 |
| 15 | Inhibition of the respiratory center | | |  |  | 1 | 2 | 1 | 0 | 4 | 2 |
| 17 | Awakening reaction | |  |  |  | 0 | 0 | 1 | 2 | 3 | 2 |
| 18 | Reduced release of dopamine in the neostriatum | | | |  | 0 | 0 | 0 | 1 | 1 | 1 |
| 19 | Increase in ganglionic transmission/excitation of the ganglia | | | | | 1 | 0 | 0 | 2 | 3 | 2 |
| 20 | Increase in cognitive performance | | |  |  | 0 | 0 | 0 | 2 | 2 | 2 |
| 21 | Presynaptic regulation of transmitter release | | | |  | 0 | 0 | 0 | 2 | 2 | 2 |
| 22 | Extrapyramidal movement coordination/tremor | | | |  | 0 | 0 | 0 | 2 | 2 | 2 |
| 48 | Activation of secretory centers of the CNS | | | |  | 2 | 0 | 0 | 0 | 2 | 2 |
|  | Total mentions | |  |  |  | 51 | 48 | 53 | 65 | 217 |  |
|  | Average range | |  |  |  |  |  |  |  |  | 1.58 |

Table S7: Category "Effects" with encodings and results for pilocarpine. The frequency with which each encoding is represented per textbook group and the encoding´s range calculated from this is shown. In addition, the average range of encodings in this category was calculated. The average range of the category is rounded to two decimal places.

| Coding | Effects | |  |  |  | 1878-1901 | 1919-1944 | 1951-1986 | 1997-2020 | All | Range |
| --- | --- | --- | --- | --- | --- | --- | --- | --- | --- | --- | --- |
|  | Cardiovascular system | |  |  |  | 11 | 10 | 8 | 7 | 36 |  |
| 1 | Negative chronotropy, inotropy or dromotropy | | | |  | 4 | 4 | 3 | 4 | 15 | 1 |
| 2 | Positive chronotropy, inotropy or dromotropy | | | |  | 4 | 2 | 1 | 0 | 7 | 4 |
| 3 | Vasodilatation | |  |  |  | 0 | 2 | 4 | 3 | 9 | 4 |
| 4 | Vasoconstriction | |  |  |  | 2 | 2 | 0 | 0 | 4 | 2 |
| 13 | Excitation of the cardiac inhibitory fibers of the vagus nerve | | | | | 1 | 0 | 0 | 0 | 1 | 1 |
|  | Eyes |  |  |  |  | 13 | 13 | 10 | 8 | 44 |  |
| 5 | Contraction of the sphincter pupillae muscle/miosis | | | |  | 4 | 4 | 4 | 4 | 16 | 0 |
| 6 | Contraction of the ciliary muscle | | |  |  | 2 | 4 | 3 | 4 | 13 | 2 |
| 7 | Contraction of the dilator pupillae muscle | | |  |  | 1 | 1 | 0 | 0 | 2 | 1 |
| 8 | Paralysis of the dilator pupillae muscle | | |  |  | 1 | 0 | 0 | 0 | 1 | 1 |
| 10 | Excitation of the oculomotor nerve | | |  |  | 2 | 0 | 0 | 0 | 2 | 2 |
| 11 | Reduction of the intraocular pressure | | |  |  | 3 | 4 | 2 | 0 | 9 | 4 |
| 12 | Inhibition of the aqueous humor formation | | | |  | 0 | 0 | 1 | 0 | 1 | 1 |
|  | Peripheral secretory processes | | |  |  | 10 | 12 | 16 | 20 | 58 |  |
| 28 | Increase in tear secretion | |  |  |  | 1 | 2 | 0 | 3 | 6 | 3 |
| 29 | Increase in saliva secretion/stimulation of the salivary secretory nerves | | | | | 4 | 3 | 4 | 4 | 15 | 1 |
| 30 | Inhibition of saliva secretion | | |  |  | 1 | 0 | 0 | 0 | 1 | 1 |
| 31 | Increase in bronchial secretion, contraction and ciliated epithelial movements | | | | | 0 | 2 | 3 | 4 | 9 | 4 |
| 32 | Increase in sweat secretion/stimulation of the sweat secretion nerves | | | | | 2 | 3 | 4 | 4 | 13 | 2 |
| 33 | Increase in tracheal secretion and contraction | | | |  | 0 | 0 | 1 | 0 | 1 | 1 |
| 35 | Increase in mucus secretion | | |  |  | 1 | 2 | 1 | 0 | 4 | 2 |
| 37 | Inhibition of secretions (no specification) | | |  |  | 1 | 0 | 0 | 0 | 1 | 1 |
| 48 | Increased insulin secretion in the pancreas | | | |  | 0 | 0 | 0 | 2 | 2 | 2 |
| 49 | Increased adrenaline release in the adrenal medulla | | | |  | 0 | 0 | 3 | 2 | 5 | 3 |
| 50 | Increased histamine release in the mast cells | | | |  | 0 | 0 | 0 | 1 | 1 | 1 |
|  | Sexual organs | |  |  |  | 3 | 2 | 2 | 1 | 8 |  |
| 38 | Contraction/variable effect on the uterus | | |  |  | 3 | 2 | 1 | 1 | 7 | 2 |
| 51 | Erection of the male sexual organs | | |  |  | 0 | 0 | 1 | 0 | 1 | 1 |
|  | Internal organs | |  |  |  | 8 | 6 | 14 | 16 | 44 |  |
| 39 | Relaxation of the sphincters of the GI tract | | | |  | 0 | 0 | 1 | 2 | 3 | 2 |
| 40 | Contraction of the bladder and detrusor vesicae muscle and increase in ureteral motility | | | | | 2 | 1 | 4 | 4 | 11 | 3 |
| 34 | Increase in peristalsis, contraction and secretion in the gastrointestinal tract | | | | | 4 | 4 | 4 | 4 | 16 | 0 |
| 41 | Relaxation of the bladder neck muscles/sphincters | | | |  | 0 | 0 | 1 | 2 | 3 | 2 |
| 42 | Contraction/secretion of the gallbladder and sphincter of Oddi | | | | | 0 | 0 | 2 | 4 | 6 | 4 |
| 43 | Relaxation of the gallbladder sphincters | | |  |  | 0 | 0 | 1 | 0 | 1 | 1 |
| 44 | Spleen contraction | |  |  |  | 2 | 1 | 0 | 0 | 3 | 2 |
| 52 | Increase in glycogen synthesis in the liver | | | |  | 0 | 0 | 1 | 0 | 1 | 1 |
|  | CNS and PNS | |  |  |  | 11 | 3 | 10 | 17 | 41 |  |
| 14 | Inhibition of the vasomotor center | | |  |  | 1 | 0 | 0 | 0 | 1 | 1 |
| 15 | Activation of the respiratory center/increased breathing | | | |  | 2 | 0 | 1 | 0 | 3 | 2 |
| 16 | Inhibition of the respiratory center | | |  |  | 2 | 1 | 1 | 0 | 4 | 2 |
| 17 | Inhibition of motor centers of the CNS | | |  |  | 1 | 0 | 0 | 0 | 1 | 1 |
| 18 | Central nervous effects (no specification) | | |  |  | 0 | 1 | 2 | 1 | 4 | 2 |
| 19 | Awakening reaction | |  |  |  | 0 | 0 | 1 | 2 | 3 | 2 |
| 20 | Reduced release of dopamine in the neostriatum | | | |  | 0 | 0 | 0 | 1 | 1 | 1 |
| 21 | Increase in ganglionic transmission/excitation of the ganglia | | | | | 2 | 0 | 4 | 4 | 10 | 4 |
| 22 | Inhibition of ganglionic transmission | | |  |  | 0 | 0 | 1 | 0 | 1 | 1 |
| 23 | Increase in cognitive performance | | |  |  | 0 | 0 | 0 | 3 | 3 | 3 |
| 24 | Presynaptic regulation of transmitter release | | | |  | 0 | 0 | 0 | 3 | 3 | 3 |
| 25 | Extrapyramidal movement coordination/tremor | | | |  | 0 | 0 | 0 | 2 | 2 | 2 |
| 26 | Reduction in motility, sensitivity and reflex response of the CNS | | | | | 1 | 0 | 0 | 0 | 1 | 1 |
| 27 | Inhibition of the CNS or its centers (no specification) | | | |  | 2 | 1 | 0 | 0 | 3 | 2 |
| 53 | Activation of the mesolimbic dopaminergic reward pathway | | | | | 0 | 0 | 0 | 1 | 1 | 1 |
|  | Musculoskeletal system | |  |  |  | 5 | 4 | 4 | 4 | 17 |  |
| 46 | Increase in neuromuscular excitation transmission/increase in muscle tone | | | | | 4 | 4 | 8 | 4 | 16 | 4 |
| 47 | Inhibition of neuromuscular transmission of excitation/lowering of muscle tone | | | | | 1 | 0 | 0 | 0 | 1 | 1 |
|  | Total mentions | |  |  |  | 61 | 50 | 64 | 73 | 248 |  |
|  | Average range | |  |  |  |  |  |  |  |  | 1.82 |

Table S8: Category "Effects" with encodings and results for physostigmine. The frequency with which each encoding is represented per textbook group and the encoding´s range calculated from this is shown. In addition, the average range of encodings in this category was calculated. The average range of the category is rounded to two decimal places.

| Coding | Indications |  |  |  | 1878-1901 | 1919-1944 | 1951-1986 | 1997-2020 | All | Range |
| --- | --- | --- | --- | --- | --- | --- | --- | --- | --- | --- |
|  | Ophthalmologic diseases | |  |  | 10 | 5 | 3 | 4 | 22 |  |
| 10 | Retinitis albuminurica | |  |  | 1 | 0 | 0 | 0 | 1 | 1 |
| 11 | Retinal detachment | |  |  | 2 | 0 | 0 | 0 | 2 | 2 |
| 12 | Glaucoma/to lower the intraocular pressure | | | | 2 | 2 | 3 | 4 | 11 | 2 |
| 13 | Vitreous opacity | |  |  | 2 | 1 | 0 | 0 | 3 | 2 |
| 14 | Eye infections | |  |  | 1 | 1 | 0 | 0 | 2 | 1 |
| 15 | Visual impairment in chronic tobacco poisoning | | | | 1 | 0 | 0 | 0 | 1 | 1 |
| 37 | Miotic agent |  |  |  | 1 | 1 | 0 | 0 | 2 | 1 |
|  | Pulmonary diseases | |  |  | 8 | 4 | 0 | 0 | 12 |  |
| 3 | Exudates and transudates | |  |  | 3 | 3 | 0 | 0 | 6 | 3 |
| 4 | Acute infections of the lungs and pleura | | |  | 2 | 0 | 0 | 0 | 2 | 2 |
| 5 | Asthma |  |  |  | 2 | 0 | 0 | 0 | 2 | 2 |
| 6 | Colds and flu |  |  |  | 1 | 1 | 0 | 0 | 2 | 1 |
|  | Gynecological and urological diseases | | |  | 3 | 0 | 1 | 0 | 4 |  |
| 1 | Amenorrhea/menstrual irregularities | | |  | 1 | 0 | 1 | 0 | 2 | 1 |
| 2 | Labor induction and obstetrics | | |  | 1 | 0 | 0 | 0 | 1 | 1 |
| 18 | Metastatic orchitis in the course of mumps | | | | 1 | 0 | 0 | 0 | 1 | 1 |
|  | Skin and sexual diseases | |  |  | 3 | 0 | 0 | 0 | 3 |  |
| 8 | Chronic skin diseases | |  |  | 1 | 0 | 0 | 0 | 1 | 1 |
| 16 | Syphilis |  |  |  | 1 | 0 | 0 | 0 | 1 | 1 |
| 19 | Erysipelas |  |  |  | 1 | 0 | 0 | 0 | 1 | 1 |
|  | Ear diseases | |  |  | 0 | 1 | 1 | 0 | 2 |  |
| 20 | Parotitis |  |  |  | 0 | 1 | 0 | 0 | 1 | 1 |
| 22 | Meniere's disease | |  |  | 0 | 0 | 1 | 0 | 1 | 1 |
|  | Kidney diseases | |  |  | 5 | 2 | 0 | 0 | 7 |  |
| 9 | Kidney diseases | |  |  | 4 | 2 | 0 | 0 | 6 | 4 |
| 38 | Diuretic agent | |  |  | 1 | 0 | 0 | 0 | 1 | 1 |
|  | Gastrointestinal diseases | |  |  | 0 | 0 | 0 | 1 | 1 |  |
| 34 | Intestinal atony | |  |  | 0 | 0 | 0 | 1 | 1 | 1 |
|  | Metabolic and autoimmune diseases | | |  | 2 | 1 | 0 | 2 | 5 |  |
| 7 | Rheumatic diseases | |  |  | 1 | 1 | 0 | 0 | 2 | 1 |
| 24 | Diabetes mellitus | |  |  | 1 | 0 | 0 | 0 | 1 | 1 |
| 32 | Sjögren´s syndrome | |  |  | 0 | 0 | 0 | 1 | 1 | 1 |
| 33 | Diagnosis of mucoviscidosis | | |  | 0 | 0 | 0 | 1 | 1 | 1 |
|  | Diseases of the cardiovascular system | | |  | 1 | 0 | 1 | 0 | 2 |  |
| 23 | Claudication intermittens | |  |  | 1 | 0 | 0 | 0 | 1 | 1 |
| 25 | Paroxysmal tachycardia | |  |  | 0 | 0 | 1 | 0 | 1 | 1 |
|  | Diseases of the nervous and skeletal system | | | | 2 | 0 | 0 | 0 | 2 |  |
| 21 | Cerebrospinal meningitis | |  |  | 1 | 0 | 0 | 0 | 1 | 1 |
| 29 | Hemianesthesia | |  |  | 1 | 0 | 0 | 0 | 1 | 1 |
|  | Intoxications |  |  |  | 3 | 1 | 3 | 0 | 7 |  |
| 26 | Belladonna intoxication | |  |  | 1 | 0 | 0 | 0 | 1 | 1 |
| 27 | Atropine/parasympatholytic intoxication | | |  | 1 | 1 | 2 | 0 | 4 | 2 |
| 28 | Botulism |  |  |  | 0 | 0 | 1 | 0 | 1 | 1 |
| 39 | Chronic metal poisoning | |  |  | 1 | 0 | 0 | 0 | 1 | 1 |
|  | Others |  |  |  | 8 | 5 | 2 | 1 | 16 |  |
| 17 | Diphtheria |  |  |  | 2 | 0 | 0 | 0 | 2 | 2 |
| 30 | Hairlessness |  |  |  | 2 | 0 | 0 | 0 | 2 | 2 |
| 31 | Foetid foot sweat | |  |  | 1 | 0 | 0 | 0 | 1 | 1 |
| 35 | To increase saliva secretion | | |  | 1 | 2 | 2 | 1 | 6 | 1 |
| 36 | Diaphoretic agent | |  |  | 2 | 3 | 0 | 0 | 5 | 3 |
|  | Total mentions | |  |  | 45 | 19 | 11 | 8 | 83 |  |
|  | Average range | |  |  |  |  |  |  |  | 1.38 |

Table S9: Category "Indications" with encodings and results for pilocarpine. The frequency with which each encoding is represented per textbook group and the encoding´s range calculated from this is shown. In addition, the average range of encodings in this category was calculated. The average range of the category is rounded to two decimal places.

| Coding | Indications | | | | 1878-1901 | 1919-1944 | 1951-1986 | 1997-2020 | All | Range |
| --- | --- | --- | --- | --- | --- | --- | --- | --- | --- | --- |
|  | Ophthalmologic diseases | |  |  | 26 | 9 | 5 | 1 | 41 |  |
| 1 | Glaucoma/reduction of intraocular pressure | | | | 4 | 4 | 4 | 1 | 13 | 3 |
| 2 | Miotic agent |  |  |  | 1 | 1 | 0 | 0 | 2 | 1 |
| 3 | Iris prolapse |  |  |  | 2 | 1 | 1 | 0 | 4 | 2 |
| 4 | Keratocele |  |  |  | 2 | 0 | 0 | 0 | 2 | 2 |
| 5 | Cornea conica | |  |  | 2 | 0 | 0 | 0 | 2 | 2 |
| 6 | Staphylomatous processes | | |  | 2 | 0 | 0 | 0 | 2 | 2 |
| 7 | Corneal ulceration | |  |  | 2 | 1 | 0 | 0 | 3 | 2 |
| 8 | Postoperative after cataract and glaucoma surgery | | | | 2 | 0 | 0 | 0 | 2 | 2 |
| 9 | Paralysis of the oculomotor nerve | | |  | 2 | 0 | 0 | 0 | 2 | 2 |
| 10 | Accommodation disorders and paralysis of the smooth eye muscles | | | | 3 | 1 | 0 | 0 | 4 | 3 |
| 11 | Solution of synechiae | |  |  | 3 | 0 | 0 | 0 | 3 | 3 |
| 12 | Preoperative before iridectomy | | |  | 0 | 1 | 0 | 0 | 1 | 1 |
| 24 | Gymnastic strengthening of the eye muscles | | | | 1 | 0 | 0 | 0 | 1 | 1 |
|  | Gastrointestinal diseases | |  |  | 2 | 2 | 0 | 0 | 4 |  |
| 16 | Intestinal atony, intestinal paralysis, flatulence and constipation | | | | 1 | 2 | 0 | 0 | 3 | 2 |
| 17 | Meteorism |  |  |  | 1 | 0 | 0 | 0 | 1 | 1 |
|  | Diseases of the nervous and skeletal system | | | | 4 | 0 | 4 | 0 | 8 |  |
| 13 | Tetanus |  |  |  | 2 | 0 | 0 | 0 | 2 | 2 |
| 14 | Spastic contractures | |  |  | 1 | 0 | 0 | 0 | 1 | 1 |
| 15 | Neonatal trismus | |  |  | 1 | 0 | 0 | 0 | 1 | 1 |
| 18 | Postdiphteric paralysis | |  |  | 0 | 0 | 1 | 0 | 1 | 1 |
| 19 | Muscle weakness following poliomyelitis | | |  | 0 | 0 | 1 | 0 | 1 | 1 |
| 20 | Amyotrophic lateral sclerosis | | |  | 0 | 0 | 1 | 0 | 1 | 1 |
| 21 | Paralysis of skeletal muscles due to curare or curare-like stabilizing muscle relaxants | | | | 0 | 0 | 1 | 0 | 1 | 1 |
|  | Intoxications |  |  |  | 2 | 1 | 2 | 5 | 10 |  |
| 22 | Anticholinergic (parasympatholytic) syndrome | | | | 0 | 0 | 0 | 1 | 1 | 1 |
| 23 | Intoxications with anticholinergic agents | | |  | 2 | 1 | 2 | 4 | 9 | 3 |
|  | Total mentions | |  |  | 34 | 12 | 11 | 6 | 63 |  |
|  | Average range | |  |  |  |  |  |  |  | 1.71 |

Table S10: Category "Indications" with encodings and results for physostigmine. The frequency with which each encoding is represented per textbook group and the encoding´s range calculated from this is shown. In addition, the average range of encodings in this category was calculated. The average range of the category is rounded to two decimal places.

| Coding | Adverse drug reactions | |  |  |  |  | 1878-1901 | 1919-1944 | 1951-1986 | 1997-2020 | All | Range |
| --- | --- | --- | --- | --- | --- | --- | --- | --- | --- | --- | --- | --- |
|  | Cardiovascular system | |  |  |  |  | 10 | 9 | 15 | 9 | 43 |  |
| 10 | Drop in blood pressure | |  |  |  |  | 3 | 1 | 4 | 2 | 10 | 3 |
| 11 | Bradycardia |  |  |  |  |  | 0 | 0 | 3 | 3 | 6 | 3 |
| 12 | Heart failure and cardiac arrest | | |  |  |  | 3 | 1 | 2 | 0 | 6 | 3 |
| 13 | Atrial fibrillation or flutter, ventricular fibrillation or flutter | | | |  |  | 0 | 0 | 2 | 0 | 2 | 2 |
| 14 | Reflective tachycardia | |  |  |  |  | 0 | 0 | 0 | 1 | 1 | 1 |
| 7 | AV block |  |  |  |  |  | 0 | 0 | 2 | 0 | 2 | 2 |
| 16 | Cardiovascular collapse | |  |  |  |  | 2 | 1 | 2 | 1 | 6 | 1 |
| 17 | Skin redness, feeling of warmth and hyperemia | | | |  |  | 1 | 3 | 0 | 1 | 5 | 3 |
| 18 | Tapping of the carotid arteries | | |  |  |  | 1 | 2 | 0 | 0 | 3 | 2 |
| 19 | Palpitation and angina pectoris | | |  |  |  | 0 | 1 | 0 | 1 | 2 | 1 |
|  | Respiratory system | |  |  |  |  | 1 | 5 | 5 | 3 | 14 |  |
| 29 | Asthmatic attacks/bronchospasm | | |  |  |  | 0 | 2 | 4 | 3 | 9 | 4 |
| 30 | Respiratory paralysis and dyspnea | | |  |  |  | 1 | 2 | 0 | 0 | 3 | 2 |
| 31 | Pulmonary edema | |  |  |  |  | 0 | 1 | 1 | 0 | 2 | 1 |
|  | Eyes |  |  |  |  |  | 5 | 4 | 6 | 6 | 21 |  |
| 1 | Disorders of near or distance vision/accommodation disorders | | | | |  | 2 | 2 | 2 | 3 | 9 | 1 |
| 2 | Spasms of the ciliary muscle/accommodation spasm | | | |  |  | 1 | 1 | 2 | 1 | 5 | 1 |
| 3 | Impaired twilight vision | |  |  |  |  | 0 | 0 | 0 | 2 | 2 | 2 |
| 4 | Foggy vision |  |  |  |  |  | 1 | 0 | 0 | 0 | 1 | 1 |
| 5 | Amblyopia |  |  |  |  |  | 1 | 0 | 0 | 0 | 1 | 1 |
| 6 | Macropia |  |  |  |  |  | 0 | 1 | 1 | 0 | 2 | 1 |
| 8 | Eyelid twitching | |  |  |  |  | 0 | 0 | 1 | 0 | 1 | 1 |
|  | Internal organs | |  |  |  |  | 12 | 9 | 9 | 12 | 42 |  |
| 26 | Spasms of the gastrointestinal tract | | |  |  |  | 0 | 1 | 2 | 1 | 4 | 2 |
| 27 | Abdominal pain and dyspepsia | | |  |  |  | 0 | 0 | 0 | 2 | 2 | 2 |
| 28 | Intensification of a peptic ulcer disease | | |  |  |  | 0 | 0 | 0 | 1 | 1 | 1 |
| 41 | Nausea and vomiting | |  |  |  |  | 4 | 3 | 3 | 3 | 13 | 1 |
| 42 | Diarrhea |  |  |  |  |  | 3 | 4 | 3 | 3 | 13 | 1 |
| 44 | Intestinal bleeding and bloody diarrhea | | |  |  |  | 0 | 1 | 0 | 0 | 1 | 1 |
| 52 | Urinary incontinence | |  |  |  |  | 0 | 0 | 1 | 0 | 1 | 1 |
| 32 | Increased diuresis and urinary urgency | | |  |  |  | 3 | 0 | 0 | 2 | 5 | 3 |
| 33 | Decreased diuresis | |  |  |  |  | 2 | 0 | 0 | 0 | 2 | 2 |
|  | Musculoskeletal system | |  |  |  |  | 3 | 3 | 0 | 0 | 6 |  |
| 47 | Muscular hyperexcitability | |  |  |  |  | 0 | 1 | 0 | 0 | 1 | 1 |
| 45 | Pain in the lumbar area | |  |  |  |  | 1 | 0 | 0 | 0 | 1 | 1 |
| 50 | Rotational movements | |  |  |  |  | 1 | 1 | 0 | 0 | 2 | 1 |
| 9 | Cramp-like twitching and trembling | | |  |  |  | 1 | 1 | 0 | 0 | 2 | 1 |
|  | CNS and PNS | |  |  |  |  | 3 | 1 | 1 | 3 | 8 |  |
| 20 | Unconsciousness and impaired consciousness | | | |  |  | 0 | 0 | 1 | 0 | 1 | 1 |
| 21 | Confusion and hallucinations | | |  |  |  | 0 | 0 | 0 | 1 | 1 | 1 |
| 22 | Mood swings | |  |  |  |  | 0 | 0 | 0 | 1 | 1 | 1 |
| 23 | Weakness and propensity to sleep | | |  |  |  | 1 | 0 | 0 | 0 | 1 | 1 |
| 24 | Headaches and dizziness | |  |  |  |  | 1 | 0 | 0 | 1 | 2 | 1 |
| 15 | Cerebral anemia | |  |  |  |  | 1 | 0 | 0 | 0 | 1 | 1 |
| 51 | Central nervous disorders (no specification) | | | |  |  | 0 | 1 | 0 | 0 | 1 | 1 |
|  | Peripheral secretory processes | | |  |  |  | 8 | 8 | 7 | 9 | 32 |  |
| 36 | Salivation |  |  |  |  |  | 4 | 3 | 2 | 3 | 12 | 2 |
| 37 | Severe sweating | |  |  |  |  | 4 | 4 | 3 | 4 | 15 | 1 |
| 38 | Lacrimation |  |  |  |  |  | 0 | 0 | 0 | 2 | 2 | 2 |
| 39 | Severe bronchial secretion | |  |  |  |  | 0 | 1 | 2 | 0 | 3 | 2 |
|  | Sexual organs | |  |  |  |  | 1 | 3 | 1 | 0 | 5 |  |
| 48 | Miscarriage |  |  |  |  |  | 1 | 2 | 1 | 0 | 4 | 2 |
| 49 | Spasms of the uterus | |  |  |  |  | 0 | 1 | 0 | 0 | 1 | 1 |
|  | Thermal and energy management | | |  |  |  | 8 | 0 | 0 | 0 | 8 |  |
| 34 | Increased body temperature | | |  |  |  | 3 | 0 | 0 | 0 | 3 | 3 |
| 35 | Decreased body temperature | | |  |  |  | 3 | 0 | 0 | 0 | 3 | 3 |
| 43 | Frosty feeling | |  |  |  |  | 1 | 0 | 0 | 0 | 1 | 1 |
| 46 | Influence on metabolism (no specification) | | | |  |  | 1 | 0 | 0 | 0 | 1 | 1 |
|  | Others |  |  |  |  |  | 0 | 0 | 0 | 1 | 1 |  |
| 25 | Allergic reactions and itchiness | | |  |  |  | 0 | 0 | 0 | 1 | 1 | 1 |
|  | Total mentions | |  |  |  |  | 51 | 42 | 44 | 43 | 180 |  |
|  | Average range | |  |  |  |  |  |  |  |  |  | 1.57 |

Table S11: Category "Adverse drug reactions" with encodings and results for pilocarpine. The frequency with which each encoding is represented per textbook group and the encoding´s range calculated from this is shown. In addition, the average range of encodings in this category was calculated. The average range of the category is rounded to two decimal places.

| Coding | Adverse drug reactions | |  |  |  | 1878-1901 | 1919-1944 | 1951-1986 | 1997-2020 | All | Range |
| --- | --- | --- | --- | --- | --- | --- | --- | --- | --- | --- | --- |
|  | Cardiovascular system | |  |  |  | 6 | 7 | 15 | 9 | 37 |  |
| 6 | Increase in blood pressure | |  |  |  | 2 | 1 | 0 | 0 | 3 | 2 |
| 7 | Drop in blood pressure | |  |  |  | 0 | 1 | 4 | 3 | 8 | 4 |
| 8 | AV block |  |  |  |  | 0 | 0 | 2 | 0 | 2 | 2 |
| 9 | Bradycardia |  |  |  |  | 0 | 0 | 3 | 4 | 7 | 4 |
| 10 | Heart failure and cardiac arrest | | |  |  | 1 | 1 | 2 | 0 | 4 | 2 |
| 11 | Palpitation |  |  |  |  | 0 | 0 | 0 | 1 | 1 | 1 |
| 12 | Anemia |  |  |  |  | 1 | 0 | 0 | 0 | 1 | 1 |
| 13 | Skin redness, feeling of warmth and hyperemia | | | |  | 0 | 3 | 0 | 0 | 3 | 3 |
| 14 | Tapping of the carotid arteries | | |  |  | 0 | 1 | 0 | 0 | 1 | 1 |
| 15 | Cardiovascular collapse | |  |  |  | 2 | 0 | 2 | 0 | 4 | 2 |
| 16 | Atrial fibrillation or flutter, ventricular fibrillation or flutter | | | |  | 0 | 0 | 2 | 1 | 3 | 2 |
|  | Respiratory system | |  |  |  | 3 | 4 | 5 | 4 | 16 |  |
| 18 | Asthmatic attacks/bronchospasm | | |  |  | 0 | 2 | 4 | 2 | 8 | 4 |
| 19 | Respiratory paralysis, dyspnea, apnea | | |  |  | 3 | 1 | 0 | 2 | 6 | 3 |
| 20 | Pulmonary edema | |  |  |  | 0 | 1 | 1 | 0 | 2 | 1 |
|  | Eyes |  |  |  |  | 7 | 4 | 5 | 4 | 20 |  |
| 1 | Disorders of near or distance vision/accommodation disorders | | | | | 3 | 1 | 1 | 2 | 7 | 2 |
| 2 | Spasms of the ciliary muscle/accommodation spasm | | | |  | 4 | 2 | 2 | 0 | 8 | 4 |
| 3 | Impaired twilight vision | |  |  |  | 0 | 0 | 0 | 2 | 2 | 2 |
| 4 | Macropia |  |  |  |  | 0 | 1 | 1 | 0 | 2 | 1 |
| 5 | Eyelid twitching | |  |  |  | 0 | 0 | 1 | 0 | 1 | 1 |
|  | Internal organs | |  |  |  | 14 | 12 | 10 | 16 | 52 |  |
| 21 | Spasms of the gastrointestinal tract |  |  |  |  | 4 | 2 | 2 | 0 | 8 | 4 |
| 23 | Spasms of the bladder | |  |  |  | 0 | 2 | 0 | 0 | 2 | 2 |
| 24 | Urinary incontinence | |  |  |  | 2 | 2 | 2 | 1 | 7 | 1 |
| 25 | Fecal incontinence | |  |  |  | 1 | 2 | 1 | 0 | 4 | 2 |
| 26 | Abdominal pain and dyspepsia | | |  |  | 0 | 0 | 0 | 3 | 3 | 3 |
| 27 | Intensification of a peptic ulcer disease | | |  |  | 0 | 0 | 0 | 1 | 1 | 1 |
| 28 | Lack of appetite and weight loss | | |  |  | 0 | 0 | 0 | 1 | 1 | 1 |
| 29 | Nausea and vomiting | |  |  |  | 3 | 2 | 2 | 4 | 11 | 2 |
| 30 | Diarrhea |  |  |  |  | 4 | 2 | 3 | 3 | 12 | 2 |
| 31 | Increased diuresis and urinary urgency | | |  |  | 0 | 0 | 0 | 3 | 3 | 3 |
|  | Musculoskeletal system | |  |  |  | 8 | 6 | 7 | 4 | 25 |  |
| 32 | Fibrillar muscle twitches, spasms, tremors and rigor | | | |  | 4 | 3 | 3 | 3 | 13 | 1 |
| 33 | Central or peripheral muscle paralysis and weakness | | | |  | 3 | 1 | 3 | 0 | 7 | 3 |
| 34 | Tetanus and convulsions | |  |  |  | 1 | 2 | 1 | 1 | 5 | 1 |
|  | CNS and PNS | |  |  |  | 3 | 2 | 2 | 7 | 14 |  |
| 35 | Unconsciousness, impaired consciousness, coma | | | |  | 1 | 0 | 1 | 0 | 2 | 1 |
| 36 | Fatigue, insomnia, nightmares | | |  |  | 0 | 0 | 0 | 1 | 1 | 1 |
| 37 | Headaches and dizziness | |  |  |  | 1 | 1 | 0 | 1 | 3 | 1 |
| 38 | Confusion, hallucinations, anxiety | | |  |  | 1 | 1 | 1 | 1 | 4 | 0 |
| 39 | Agitation/aggressiveness | |  |  |  | 0 | 0 | 0 | 1 | 1 | 1 |
| 40 | Mood swings | |  |  |  | 0 | 0 | 0 | 1 | 1 | 1 |
| 17 | Depolarization block | |  |  |  | 0 | 0 | 0 | 2 | 2 | 2 |
|  | Peripheral secretory processes | | |  |  | 5 | 6 | 7 | 9 | 27 |  |
| 42 | Salivation |  |  |  |  | 2 | 4 | 3 | 3 | 12 | 2 |
| 43 | Severe sweating | |  |  |  | 2 | 2 | 3 | 3 | 10 | 1 |
| 44 | Lacrimation |  |  |  |  | 1 | 0 | 0 | 1 | 2 | 1 |
| 45 | Severe bronchial secretion | |  |  |  | 0 | 0 | 0 | 2 | 2 | 2 |
| 46 | Excessive increase in secretory processes (no specification) | | | | | 0 | 0 | 1 | 0 | 1 | 1 |
|  | Sexual organs | |  |  |  | 1 | 2 | 0 | 0 | 3 |  |
| 22 | Spasms of the uterus | |  |  |  | 0 | 1 | 0 | 0 | 1 | 1 |
| 47 | Miscarriage |  |  |  |  | 1 | 1 | 0 | 0 | 2 | 1 |
|  | Others |  |  |  |  | 0 | 0 | 0 | 1 | 1 |  |
| 41 | Allergic reactions and itchiness |  |  |  |  | 0 | 0 | 0 | 1 | 1 | 1 |
|  | Total mentions | |  |  |  | 47 | 43 | 51 | 54 | 195 |  |
|  | Average range | |  |  |  |  |  |  |  |  | 1.81 |

Table S12: Category "Adverse drug reactions" with encodings and results for physostigmine. The frequency with which each encoding is represented per textbook group and the encoding´s range calculated from this is shown. In addition, the average range of encodings in this category was calculated. The average range of the category is rounded to two decimal places.

| Coding | Interactions | | | 1878-1901 | 1919-1944 | 1951-1986 | 1997-2020 | All | Range |
| --- | --- | --- | --- | --- | --- | --- | --- | --- | --- |
| 1 | Atropine |  |  | 4 | 3 | 2 | 1 | 10 | 3 |
| 2 | Barbiturates |  |  | 0 | 0 | 1 | 0 | 1 | 1 |
| 3 | Chlorpromazine derivatives | |  | 0 | 0 | 1 | 0 | 1 | 1 |
| 4 | Parasympathomimetics (no specification) | | | 0 | 1 | 1 | 1 | 3 | 1 |
| 5 | Parasympatholytics (no specification) | | | 0 | 0 | 1 | 2 | 3 | 2 |
| 6 | ß-receptor antagonists | |  | 0 | 0 | 0 | 2 | 2 | 2 |
| 7 | Opioid analgesics | |  | 0 | 0 | 0 | 1 | 1 | 1 |
| 8 | Not specified |  |  | 0 | 1 | 1 | 0 | 2 | 1 |
|  | Total mentions | |  | 4 | 4 | 6 | 7 | 21 |  |
|  | Average range | |  |  |  |  |  |  | 1.50 |

Table S13: Category "Interactions" with encodings and results for pilocarpine. The frequency with which each encoding is represented per textbook group and the encoding´s range calculated from this is shown. In addition, the average range of encodings in this category was calculated. The average range of the category is rounded to two decimal places.

| Coding | Interactions | | | | | 1878-1901 | 1919-1944 | 1951-1986 | 1997-2020 | All | Range |
| --- | --- | --- | --- | --- | --- | --- | --- | --- | --- | --- | --- |
| 1 | Atropine |  |  |  |  | 4 | 4 | 4 | 2 | 14 | 2 |
| 2 | Anticholinergic substances (no specification) | | | |  | 0 | 0 | 0 | 1 | 1 | 1 |
| 3 | Pilocarpine |  |  |  |  | 0 | 1 | 0 | 0 | 1 | 1 |
| 4 | Alkyl phosphates | |  |  |  | 0 | 0 | 1 | 0 | 1 | 1 |
| 5 | Parasympathomimetics (no specification) | | | |  | 0 | 0 | 0 | 1 | 1 | 1 |
| 6 | Chlorpromazine derivatives | |  |  |  | 0 | 0 | 1 | 0 | 1 | 1 |
| 7 | Curare/non-depolarizing muscle relaxants (no specification) | | | | | 3 | 2 | 2 | 2 | 9 | 1 |
| 8 | Depolarizing muscle relaxants (no specification) | | | |  | 0 | 0 | 0 | 1 | 1 | 1 |
| 9 | Cocaine/novocaine | |  |  |  | 0 | 1 | 0 | 0 | 1 | 1 |
| 10 | Potassium salts | |  |  |  | 0 | 1 | 0 | 0 | 1 | 1 |
| 11 | Barbiturates |  |  |  |  | 0 | 0 | 1 | 0 | 1 | 1 |
| 12 | ß-receptor antagonists | |  |  |  | 0 | 0 | 0 | 2 | 2 | 2 |
|  | Total mentions | |  |  |  | 7 | 9 | 9 | 9 | 34 |  |
|  | Average range | |  |  |  |  |  |  |  |  | 1.17 |

Table S14: Category "Interactions" with encodings and results for physostigmine. The frequency with which each encoding is represented per textbook group and the encoding´s range calculated from this is shown. In addition, the average range of encodings in this category was calculated. The average range of the category is rounded to two decimal places.

| Coding | Contraindications | | | | | 1878-1901 | 1919-1944 | 1951-1986 | 1997-2020 | All | Range |
| --- | --- | --- | --- | --- | --- | --- | --- | --- | --- | --- | --- |
| 1 | Pulmonary edema/disposition to pulmonary edema | | | |  | 2 | 1 | 0 | 0 | 3 | 2 |
| 2 | Asthma/obstructive airway diseases | | |  |  | 0 | 1 | 3 | 3 | 7 | 3 |
| 3 | Tendency to catch colds | |  |  |  | 1 | 0 | 0 | 0 | 1 | 1 |
| 4 | Spasms of the gastrointestinal tract | | |  |  | 0 | 0 | 1 | 1 | 2 | 1 |
| 5 | Spasms of the biliary tract | |  |  |  | 0 | 0 | 1 | 1 | 2 | 1 |
| 6 | Spasms of the urinary tract | | |  |  | 0 | 0 | 1 | 1 | 2 | 1 |
| 7 | Ileus |  |  |  |  | 0 | 0 | 0 | 1 | 1 | 1 |
| 8 | Gastric ulcers | |  |  |  | 0 | 0 | 0 | 2 | 2 | 2 |
| 9 | Intestinal ulcers | |  |  |  | 0 | 0 | 0 | 1 | 1 | 1 |
| 10 | Bradycardia |  |  |  |  | 0 | 0 | 1 | 0 | 1 | 1 |
| 11 | Hypotension |  |  |  |  | 0 | 0 | 1 | 0 | 1 | 1 |
| 12 | Myocardial infarction | |  |  |  | 0 | 0 | 1 | 1 | 2 | 1 |
| 13 | Cardiac insufficiency | |  |  |  | 1 | 0 | 2 | 1 | 4 | 2 |
| 14 | Coronary insufficiency | |  |  |  | 0 | 0 | 0 | 1 | 1 | 1 |
| 15 | Parkinsonism | |  |  |  | 0 | 0 | 1 | 1 | 2 | 1 |
| 16 | Hyperthyroidism and thyrotoxicosis | | |  |  | 0 | 0 | 1 | 3 | 4 | 3 |
| 17 | Iritis |  |  |  |  | 0 | 0 | 0 | 1 | 1 | 1 |
| 18 | Pregnancy |  |  |  |  | 0 | 2 | 1 | 0 | 3 | 2 |
| 19 | No reaction to pilocarpine drops/rigid pupil | | | |  | 0 | 0 | 0 | 1 | 1 | 1 |
| 20 | Diseases in which increased secretions are contraindicated (no specification) | | | | | 1 | 1 | 0 | 0 | 2 | 1 |
| 21 | Not specified |  |  |  |  | 2 | 1 | 0 | 0 | 3 | 2 |
|  | Total mentions | |  |  |  | 5 | 5 | 14 | 19 | 43 |  |
|  | Average range | |  |  |  |  |  |  |  |  | 1.43 |

Table S15: Category "Contraindications" with encodings and results for pilocarpine. The frequency with which each encoding is represented per textbook group and the encoding´s range calculated from this is shown. In addition, the average range of encodings in this category was calculated. The average range of the category is rounded to two decimal places.

| Coding | Contraindications | | | | | 1878-1901 | 1919-1944 | 1951-1986 | 1997-2020 | All | Range |
| --- | --- | --- | --- | --- | --- | --- | --- | --- | --- | --- | --- |
| 1 | Epilepsy |  |  |  |  | 0 | 0 | 0 | 1 | 1 | 1 |
| 2 | Asthma/obstructive airway diseases | | |  |  | 0 | 1 | 3 | 3 | 7 | 3 |
| 3 | Hepatic insufficiency | |  |  |  | 0 | 0 | 0 | 1 | 1 | 1 |
| 4 | Spasms of the gastrointestinal tract | | |  |  | 0 | 0 | 1 | 1 | 2 | 1 |
| 5 | Spasms of the biliary tract | |  |  |  | 0 | 0 | 1 | 1 | 2 | 1 |
| 6 | Stenoses/spasms of the urinary tract | | |  |  | 0 | 0 | 1 | 2 | 3 | 2 |
| 7 | Ileus |  |  |  |  | 0 | 0 | 0 | 2 | 2 | 2 |
| 8 | Gastric ulcers | |  |  |  | 0 | 0 | 0 | 2 | 2 | 2 |
| 9 | Duodenal ulcers | |  |  |  | 0 | 0 | 0 | 1 | 1 | 1 |
| 10 | Bradycardia |  |  |  |  | 0 | 0 | 1 | 2 | 3 | 2 |
| 11 | Hypotension |  |  |  |  | 0 | 0 | 1 | 1 | 2 | 1 |
| 12 | Myocardial infarction | |  |  |  | 0 | 0 | 1 | 2 | 3 | 2 |
| 13 | Cardiac insufficiency | |  |  |  | 0 | 0 | 2 | 2 | 4 | 2 |
| 14 | QRS complex widening | |  |  |  | 0 | 0 | 0 | 1 | 1 | 1 |
| 15 | Cardiovascular shock | |  |  |  | 0 | 0 | 0 | 1 | 1 | 1 |
| 16 | Parkinsonism | |  |  |  | 0 | 0 | 1 | 1 | 2 | 1 |
| 17 | Hyperthyroidism and thyrotoxicosis | | |  |  | 0 | 0 | 1 | 3 | 4 | 3 |
| 18 | Iritis |  |  |  |  | 0 | 0 | 0 | 1 | 1 | 1 |
| 19 | Pregnancy and breastfeeding | | |  |  | 0 | 1 | 0 | 1 | 2 | 1 |
| 20 | Myotonia |  |  |  |  | 0 | 0 | 0 | 1 | 1 | 1 |
| 21 | Not specified |  |  |  |  | 4 | 3 | 1 | 1 | 9 | 3 |
|  | Total mentions | |  |  |  | 0 | 2 | 13 | 30 | 45 |  |
|  | Average range | |  |  |  |  |  |  |  |  | 1.57 |

Table S16: Category "Contraindications" with encodings and results for physostigmine. The frequency with which each encoding is represented per textbook group and the encoding´s range calculated from this is shown. In addition, the average range of encodings in this category was calculated. The average range of the category is rounded to two decimal places.


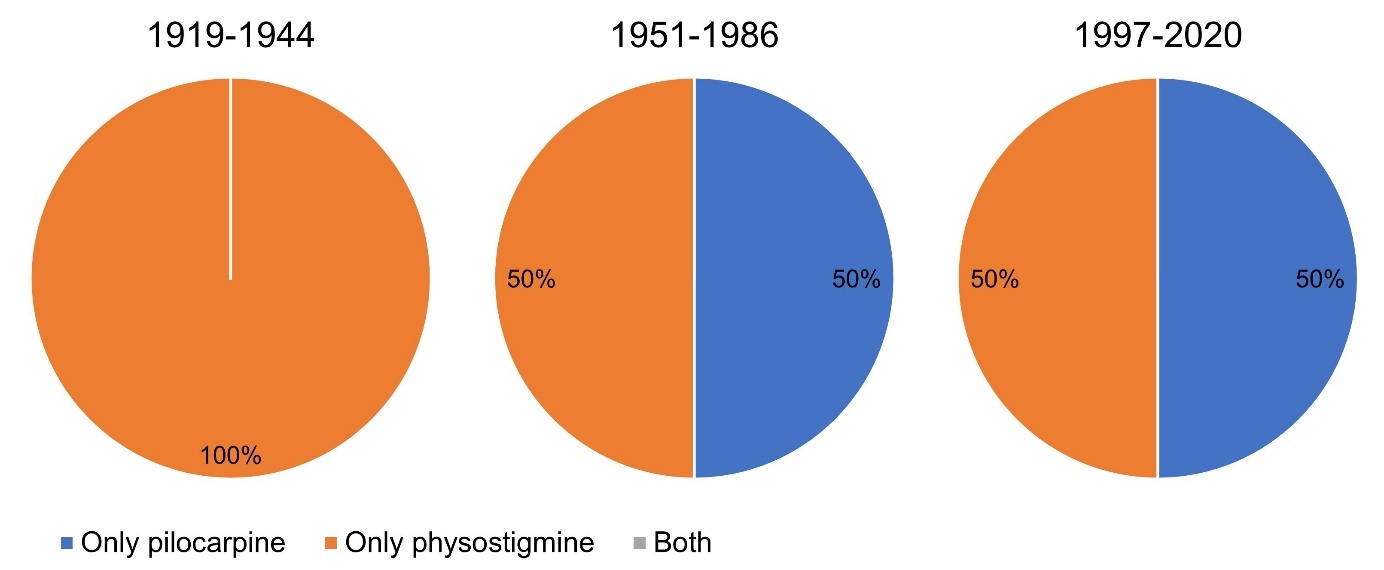


Figure S1: Occurrence of textbook contents in the category *Molecular mechanism of action*. It was analyzed which mechanisms of action were named exclusively for pilocarpine, exclusively for physostigmine or for both drugs. The data is presented as a percentage of the total number of molecular mechanisms of action mentioned. As there was no content on the molecular mechanism of action for both drugs in the first textbook group, no analysis can be made for this period. Since the second textbook group only lists content on the molecular mechanism of action for physostigmine, no content can be assigned to the category ´´only pilocarpine´´ in this period. The calculation is based on the encodings listed for pilocarpine and physostigmine and the overlap of encodings that occur for both drugs**.** The encoding *Not specified* was not integrated into the evaluation, as only content listed in the textbooks was compared.


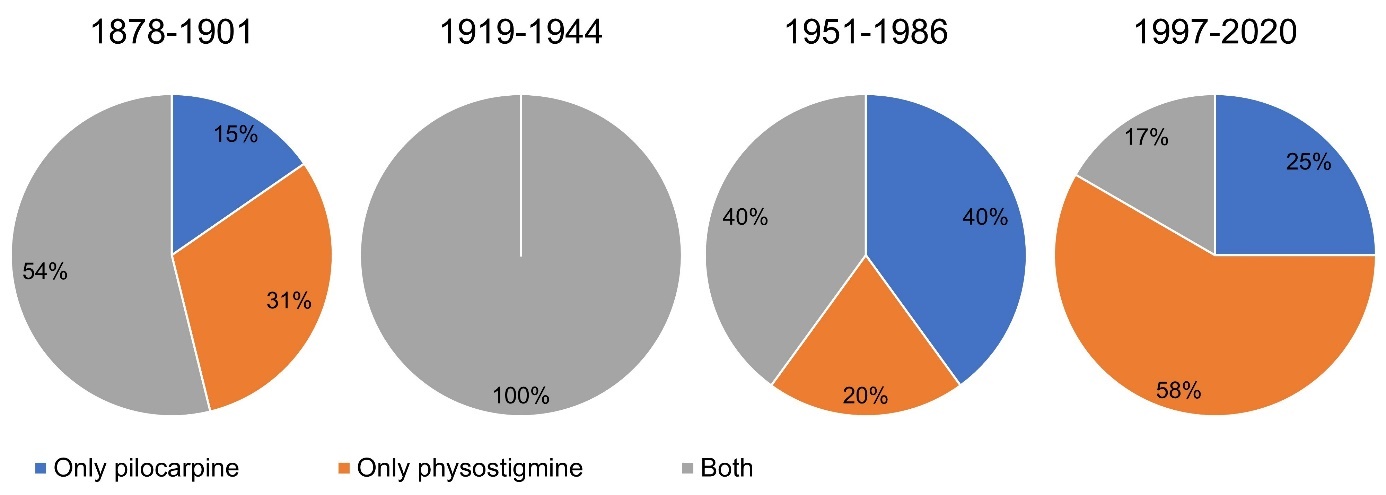


Figure S2: Occurrence of textbook contents in the category *Pharmacokinetics*. It was analyzed which drug effects were named exclusively for pilocarpine, exclusively for physostigmine or for both drugs. The data is presented as a percentage of the total number of effects mentioned. The calculation is based on the encodings listed for pilocarpine and physostigmine and the overlap of encodings that occur for both drugs. The encoding *Not specified* was not integrated into the evaluation, as only content listed in the textbooks was compared.


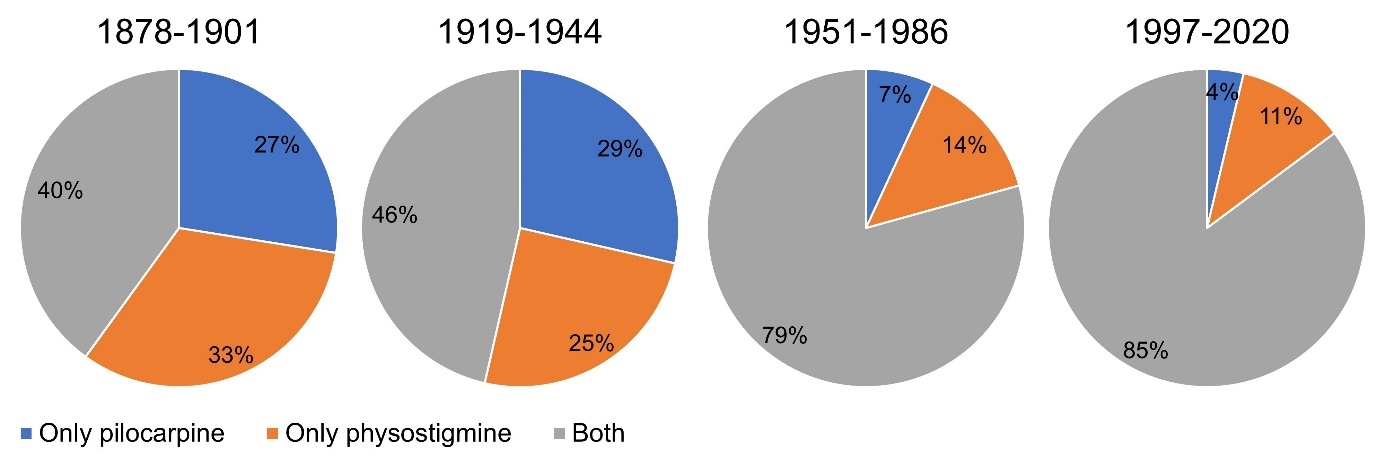


Figure S3: Occurrence of textbook contents in the category *Effects*. It was analyzed which drug effects were named exclusively for pilocarpine, exclusively for physostigmine or for both drugs. The data is presented as a percentage of the total number of effects mentioned. The calculation is based on the encodings listed for pilocarpine and physostigmine and the overlap of encodings that occur for both drugs. The encoding *Not specified* was not integrated into the evaluation, as only content listed in the textbooks was compared.


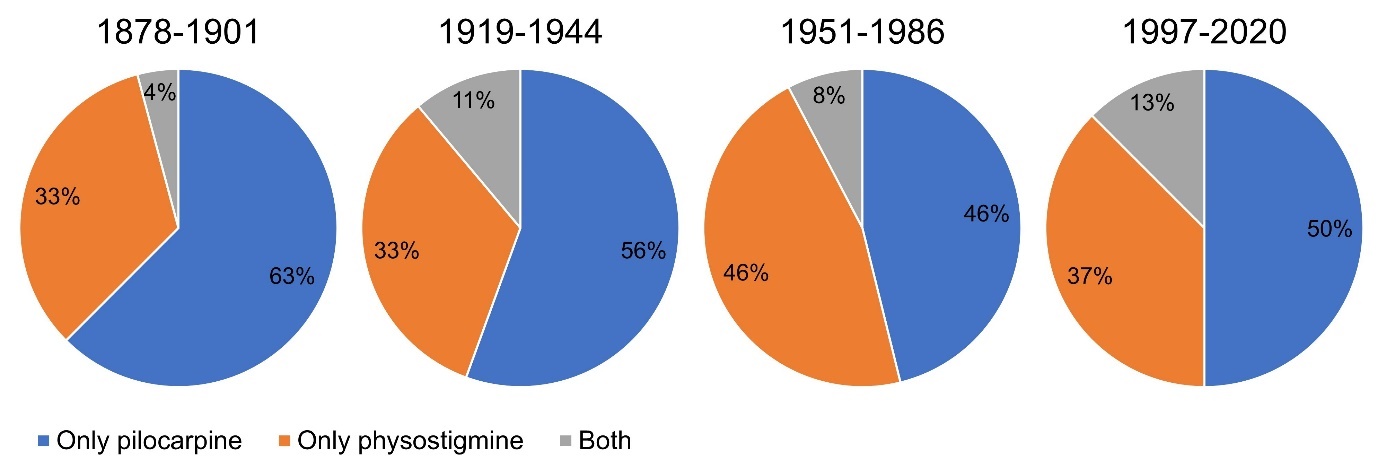


Figure S4: Occurrence of textbook contents in the category *Indications*. It was analyzed which indications were named exclusively for pilocarpine, exclusively for physostigmine or for both drugs. The data is presented as a percentage of the total number of indications mentioned. The calculation is based on the encodings listed for pilocarpine and physostigmine and the overlap of encodings that occur for both drugs. The encoding *Not specified* was not integrated into the evaluation, as only content listed in the textbooks was compared.


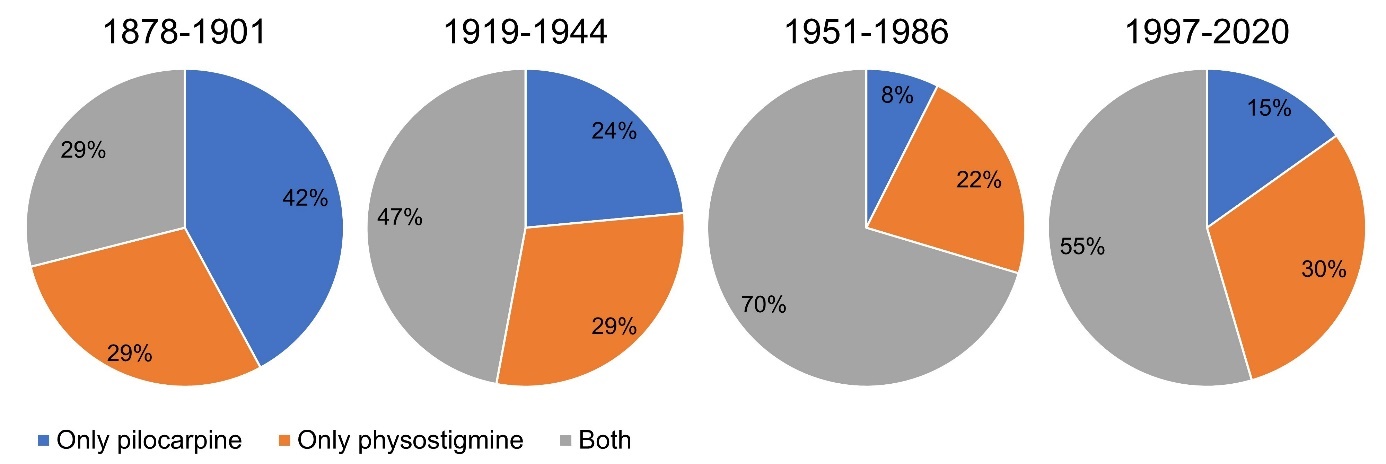


Figure S5: Occurrence of textbook contents in the category *Adverse drug reactions*. It was analyzed which adverse drug reactions were named exclusively for pilocarpine, exclusively for physostigmine or for both drugs. The data is presented as a percentage of the total number of adverse drug reactions mentioned. The calculation is based on the encodings listed for pilocarpine and physostigmine and the overlap of encodings that occur for both drugs. The encoding *Not specified* was not integrated into the evaluation, as only content listed in the textbooks was compared.


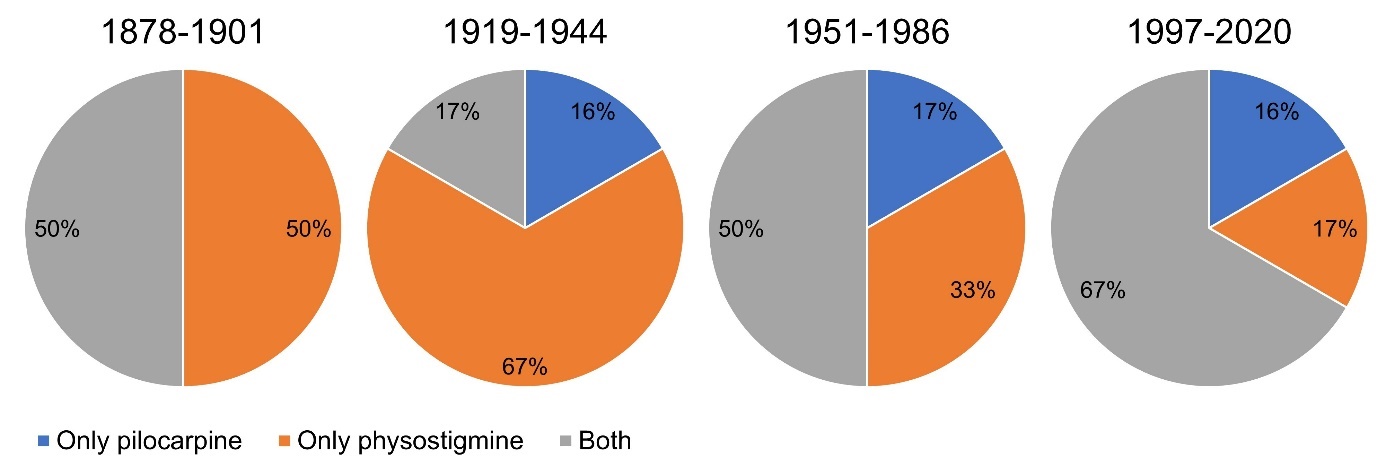


Figure S6: Occurrence of textbook contents in the category *Interactions*. It was analyzed which interactions were named exclusively for pilocarpine, exclusively for physostigmine or for both drugs. The data is presented as a percentage of the total number of interactions mentioned. The calculation is based on the encodings listed for pilocarpine and physostigmine and the overlap of encodings that occur for both drugs. The encoding *Not specified* was not integrated into the evaluation, as only content listed in the textbooks was compared.


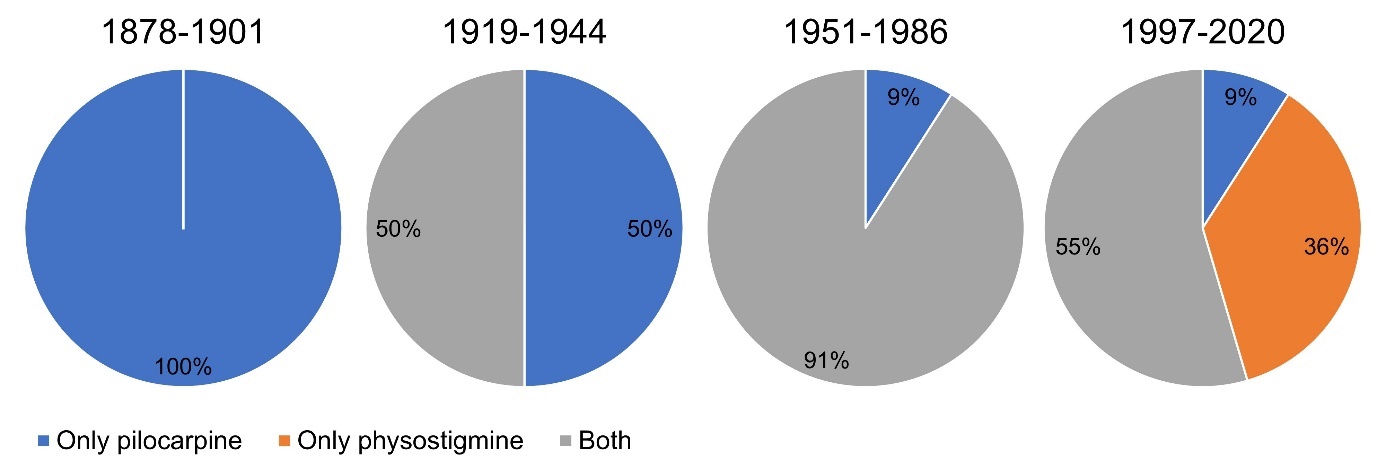


Figure S7: Occurrence of textbook contents in the category *Contraindications*. It was analyzed which contraindications were named exclusively for pilocarpine, exclusively for physostigmine or for both drugs. The data is presented as a percentage of the total number of contraindications mentioned. The calculation is based on the encodings listed for pilocarpine and physostigmine and the overlap of encodings that occur for both drugs. The encoding *Not specified* was not integrated into the evaluation, as only content listed in the textbooks was compared.


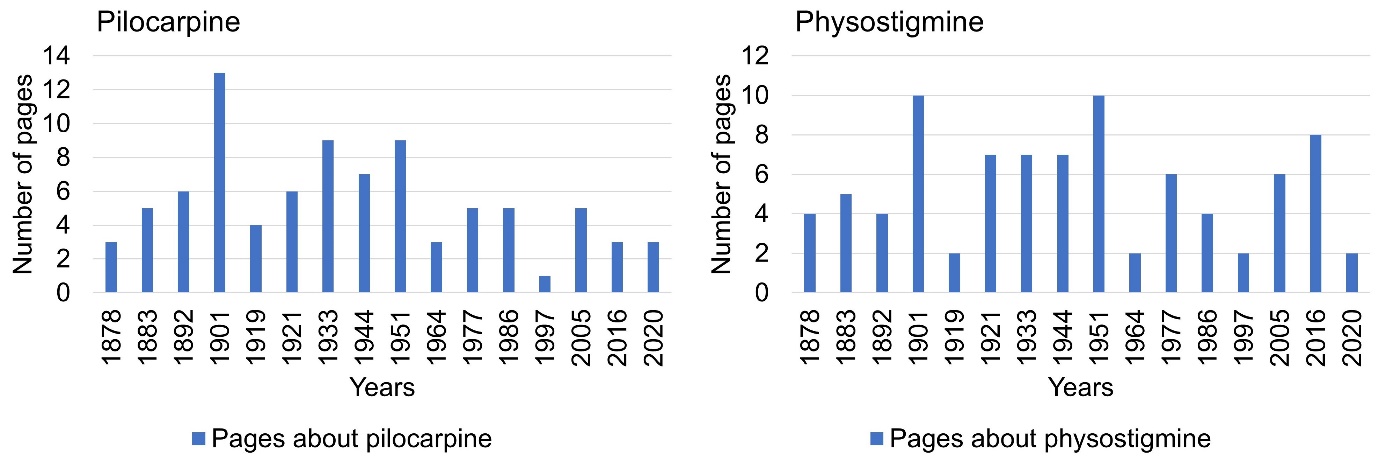


Figure S8: Number of pages with content on the active ingredients.

Panel A shows the number of pages with content on pilocarpine, panel B shows the number of pages on physostigmine. The number of pages with content on the active substance is based on the page information from the index, which relates to pilocarpine or physostigmine and is listed in Table A. If a page is listed in the index, but pilocarpine or physostigmine is not explicitly discussed on the corresponding page, the page reference is not listed in the table and included in the analysis. Pages on which content about the analyzed active substance is continued from previous or surrounding specified pages are also listed as individual pages in the table and counted in the analysis. This also applies to pages that are labelled as subsequent pages in the index.


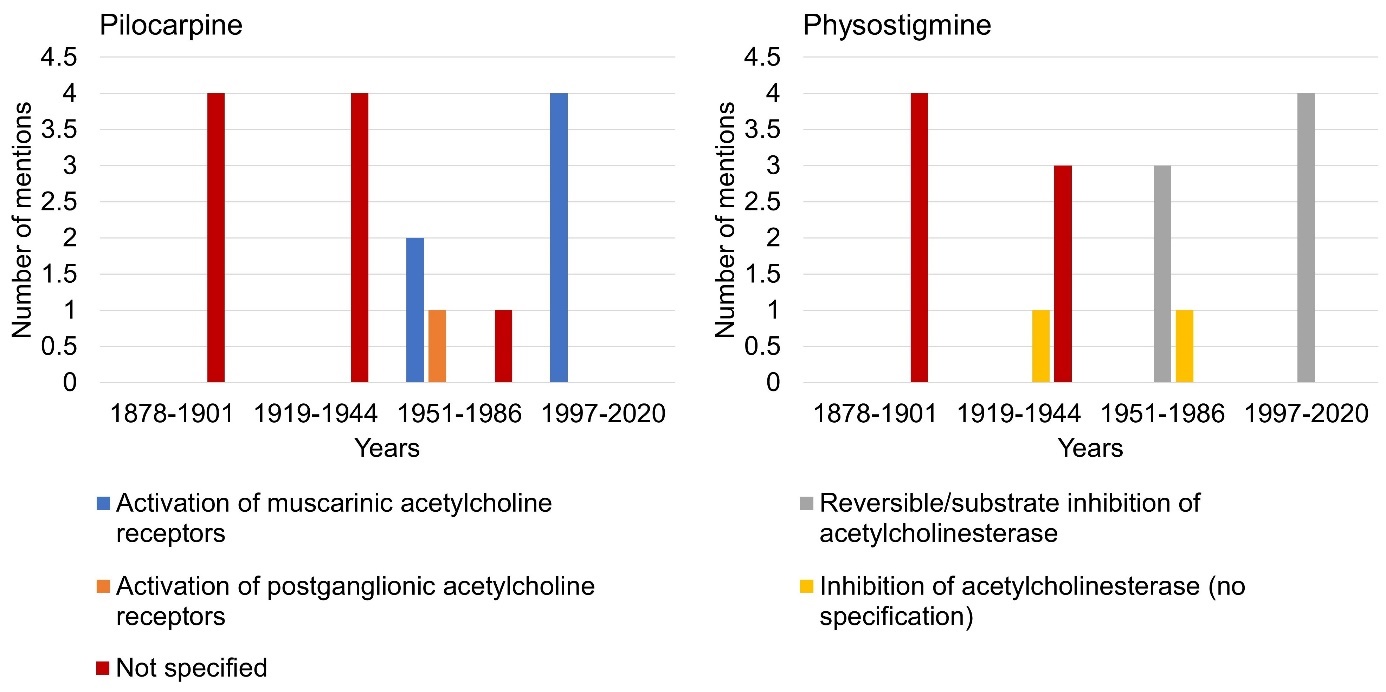


Figure S9: Information on the molecular mechanism of action of the active substances. The information for pilocarpine is shown in panel A, the entries for physostigmine are shown in panel B. The absolute amount of data is shown.

Figure S10: Total number of indications mentioned for pilocarpine and physostigmine per textbook group. The indications mentioned for pilocarpine and physostigmine are shown combined. An exponential regression was generated.

Figure S11: Number of adverse drug reactions mentioned for pilocarpine and physostigmine according to individual textbooks.

Figure S12: Number of Google Scholar search results for pilocarpine and physostigmine. The number of pilocarpine and physostigmine entries per year for the study period 1878-2020 is shown. No filters were applied during the search. Patents and citations were not included in the survey. Due to the increased inclusion of predatory content in the Google Scholar search results and thus reduced scientific reliability, the data listed here is evaluated exclusively as a supplement to the PubMed search results in figure 16.
